# Recent deep learning-based brain tumor segmentation models using multi-modality magnetic resonance imaging: a prospective survey

**DOI:** 10.3389/fbioe.2024.1392807

**Published:** 2024-07-22

**Authors:** Zain Ul Abidin, Rizwan Ali Naqvi, Amir Haider, Hyung Seok Kim, Daesik Jeong, Seung Won Lee

**Affiliations:** ^1^ Department of Intelligent Mechatronics Engineering, Sejong University, Seoul, Republic of Korea; ^2^ College of Convergence Engineering, Sangmyung University, Seoul, Republic of Korea; ^3^ School of Medicine, Sungkyunkwan University, Suwon, Republic of Korea

**Keywords:** deep learning, brain tumor segmentation, medical images, multi-modality analysis, vision transformers, convolutional neural network

## Abstract

Radiologists encounter significant challenges when segmenting and determining brain tumors in patients because this information assists in treatment planning. The utilization of artificial intelligence (AI), especially deep learning (DL), has emerged as a useful tool in healthcare, aiding radiologists in their diagnostic processes. This empowers radiologists to understand the biology of tumors better and provide personalized care to patients with brain tumors. The segmentation of brain tumors using multi-modal magnetic resonance imaging (MRI) images has received considerable attention. In this survey, we first discuss multi-modal and available magnetic resonance imaging modalities and their properties. Subsequently, we discuss the most recent DL-based models for brain tumor segmentation using multi-modal MRI. We divide this section into three parts based on the architecture: the first is for models that use the backbone of convolutional neural networks (CNN), the second is for vision transformer-based models, and the third is for hybrid models that use both convolutional neural networks and transformer in the architecture. In addition, in-depth statistical analysis is performed of the recent publication, frequently used datasets, and evaluation metrics for segmentation tasks. Finally, open research challenges are identified and suggested promising future directions for brain tumor segmentation to improve diagnostic accuracy and treatment outcomes for patients with brain tumors. This aligns with public health goals to use health technologies for better healthcare delivery and population health management.

## 1 Introduction

### 1.1 Background

The brain contains around one hundred billion neurons and is an essential organ in the human body (Stiles and Jernigan, 2010). Brain and other nervous system tumors are a significant cause of mortality in developed nations, ranking 10th among the leading causes of death (Siegel et al., 2023). This condition impacts individuals throughout various age groups, including adults and children. According to estimates, the United States witnessed approximately 18,280 fatalities in 2022 due to primary brain tumors (Tahir et al., 2022). The brain comprises various cell types with individual characteristics, rendering generalizations concerning malignancies in other organs irrelevant (Charles et al., 2011). Common symptoms of brain tumors frequently encompass feelings of high blood pressure, severe fatigue, nausea attacks, physical discomfort with fever, skin eruptions, and increased cardiac pulsations. Although professionals attempt to establish a correlation between symptoms and a definitive diagnosis, it is essential to note that brain tumors do not consistently exhibit observable symptoms (Desjardins et al., 2019; Kotia et al., 2020).

Over the last few decades, researchers have conducted comprehensive fundamental research on brain tumors (Rao and Karunakara, 2021; Dhole and Dixit, 2022; Jyothi and Singh, 2023). The primary objective of this research is to understand biological properties and their transformation into malignant tumors. Over time, there has been significant progress in comprehending the genetic and molecular changes associated with brain tumors. This has significantly contributed to advancing novel methods for diagnosing and treating brain tumors. Additionally, researchers have explored the use of several imaging modalities, such as magnetic resonance imaging (MRI), to aid in identifying brain tumors and tracking their subsequent development (Guo et al., 2020; Yang et al., 2021). Due to its exceptional accuracy and clarity, MRI has emerged as the primary method for examining brain tumors. Consequently, this technological advancement has paved the way for innovative surgical techniques, including minimally invasive procedures (Privitera et al., 2022). These technological breakthroughs facilitate the accurate removal of brain tumors while minimizing damage to surrounding tissues. To be more specific, the primary objective of segmenting brain tumors is to accurately delineate different areas of tumors by modifying the representations obtained from MRI. The segmentation outcomes are subsequently applied to the prognosis and prediction of survival for brain malignancies.

**FIGURE 1:**
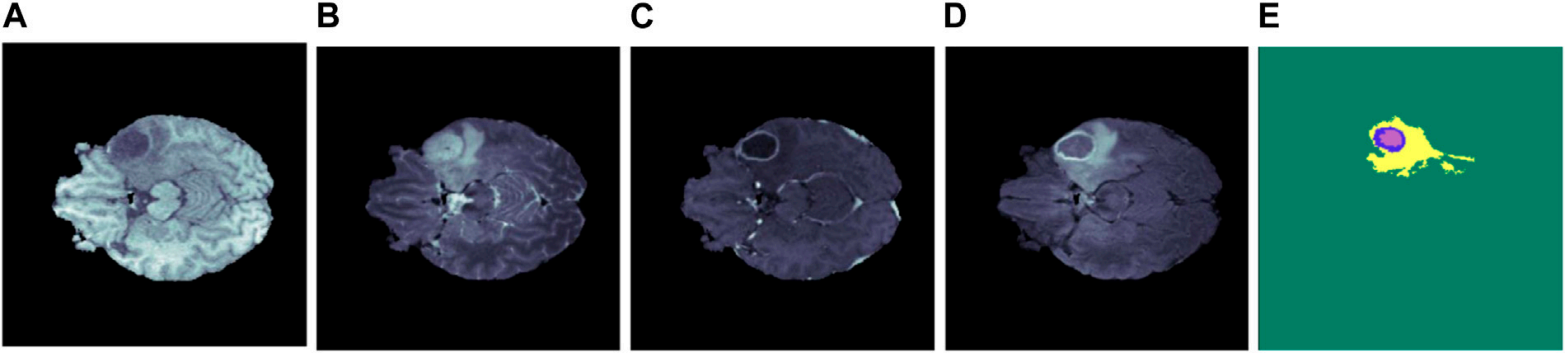
Illustration of several brain MRI modalities **(A)** T1, **(B)** T2, **(C)** T1ce, **(D)** FLAIR, and **(E)** ground truth. The yellow, blue, and purple colors in the ground truth represent edema, enhancing, and necrosis, respectively.

The broad use of multi-modal MRI images in the segmentation of brain tumors has been facilitated by advancements in MRI technology. This method provides a detailed interpretation of the tumors and the neighboring tissues. MRI includes four unique modalities: T1-weighted (T1), T2-weighted (T2), T1-weighted with contrast enhancement (T1ce), and fluid attenuation inversion recovery (FLAIR). These modalities provide extra information for diagnosing and monitoring brain tumors (Menze et al., 2014). Table 1 provides a detailed overview of these modalities along with their properties, and Figure 1 shows the MRI modalities of brain tumors. The T1 is frequently utilized to generate high-resolution brain images. On the other hand, T2 is useful for evaluating the fluid content in tissues, which serves as a key differentiator between tumors and healthy brain tissue. Additionally, the T1ce provides relevant details on the vascular structures and the enhancing characteristics of tumors, hence facilitating the classification of tumor types (Wang et al., 2023a).

**TABLE 1:** Overview of MRI modalities.

Modality	Properties
T1	• T1 images provide good anatomical detail • Highlights differences in tissue composition • Sensitive to variations in proton density and T1 relaxation times • Brighter tissue denotes shorter relaxation time
T1ce	• T1ce images acquired after administering a contrast agent (e.g., gadolinium) • Contrast agent enhances regions with the disrupted blood-brain barrier • Helps identify areas of increased vascularity
T2	• T2 images are sensitive to variations in proton density and T2 relaxation times • Emphasizes differences in tissue water content • Good for highlighting edema and lesions • Longer relaxation time is associated with brighter tissues
FLAIR	• Designed to suppress the signal from cerebrospinal fluid (CSF) • Particularly useful for highlighting abnormalities in white matter and gray matter • Minimizes the signal from the fluid

Integrating several MRI modalities provides a comprehensive and accurate depiction of tumors and adjacent brain tissue, which is essential for successful segmentation (Salvador et al., 2019). By employing multi-modal MRI images, researchers can assess the efficacy of various segmentation algorithms and make comparisons of their outcomes. This comparative study aims to stimulate the development of novel methodologies and improve the precision of brain tumor segmentation. The Brain Tumor Segmentation (BraTS) Challenge dataset is generally recognized as the principal resource for assessing brain tumor segmentation (Menze et al., 2014). The dataset consists of a wide array of MRI modalities, such as T1, T2, T1ce, and FLAIR, accompanied by precisely annotated tumor segmentation masks. The BraTS dataset is a significant resource for academics and clinicians involved in segmenting gliomas and diagnosing brain tumors.

Recent advancements in deep learning (DL) have significantly enhanced the capabilities of computer-aided analysis in various domains. In particular, the segmentation of multi-modal brain tumors has witnessed substantial progress, offering a plethora of techniques with varying degrees of accuracy and effectiveness (Rao and Karunakara, 2021; Dhole and Dixit, 2022; Jyothi and Singh, 2023). Initially, brain tumor segmentation relied on manual tracking, where skilled practitioners manually delineated tumor boundaries on medical images. However, this method is time-consuming and prone to inter-observer variability. As computer vision gains the limelight, automatic ways to separate brain tumors have become more popular. There are two main groups of these methods: traditional and DL methods. Some examples of traditional methods are atlas-based segmentation and region-growing level-set methods (Hamamci et al., 2011; Hamamci et al., 2012). Such approaches employ things about the image, like sharpness, color, etc., to segment the tumor from the surrounding tissue.

DL methods, especially convolutional neural networks (CNNs), have gotten much attention lately for how well they work at segmenting brain tumors than traditional methods. Traditional models estimate the tumor borders and locations using statistical learning techniques (Ilhan and Ilhan, 2017; Biratu et al., 2021; Khilkhal et al., 2022; Nyo et al., 2022). These models rely on preprocessing techniques to improve the quality and clarity of the tumor images before the tumor lesions are delineated. Traditional models use these techniques to help with the later investigation, characterization of the tumor, and precise estimations of tumor boundaries. On the other hand, CNN leverages DL techniques to autonomously learn hierarchical representations of features directly from the data (Pereira et al., 2016). This enables CNNs to adapt and optimize their performance based on the specific characteristics of brain tumor images, ultimately leading to more precise and reliable segmentation results compared to traditional approaches. Recently, vision transformers have made amazing progress and are now better at separating brain tumors into their different parts (Liu et al., 2022a; He et al., 2022). Some researchers have employed transformer layers that integrate a self-attention mechanism featuring multiple heads, aiming to capture additional distinctive global characteristics. Meanwhile, other researchers have devised transformer-based modules for modal fusion, facilitating the alignment of multi-modal inputs and enhancing the integration of diverse data types. This approach simplifies the process of segregating multi-modal MRI data. The objective of these studies is to discover more effective methods for visualizing multi-modal brain tumors and leveraging appropriate data to enhance tumor segmentation outcomes.

In this section, we highlight the significance of brain tumor segmentation through statistical insights. The discussion then shifts toward the importance of research conducted in the last decade and the use of MRI. Following this, we explored various modalities of MRI, such as T1, T2, T1ce, and FLAIR, along with their properties and utilization of these modalities in brain tumor segmentation. In the end, we explored the various techniques used in brain tumor segmentation and the superiority of DL-based methods. Acknowledging the complexities associated with segmenting multi-modal brain MRI due to inherent challenges, the ultimate objective of the study becomes clearer: to provide a comprehensive overview of recent DL-based models from 2021 to 2023 designed to segment brain tumor lesions in multi-modal MRI autonomously. The list of all used abbreviations is summarized in Table 2.

**TABLE 2:** List of abbreviations.

Abbreviation	Full form	Abbreviation	Full form	Abbreviation	Full form
AI	Artificial Intelligence	DL	Deep Learning	MRI	Magnetic Resonance Imaging
FLAIR	Fluid Attenuation Inversion Recovery	T1	T1-weighted	T1ce	T1-weighted with contrast enhancement
T2	T2-weighted	BraTS	Brain Tumor Segmentation	CNN	Convolutional Neural Network
ViT	Vision Transformer	MAAB	Multiple Atrous Convolutions Attention Block	MM-BiFPN	Multimodal Fusion Bi-directional Feature Pyramid Network
CMFT	Cross-Modality Feature Transition	CMFF	Cross-Modality Feature Fusion	FeG	Feature-enhanced Generator
CC	Correlation Constraints	RFNet	Region-aware Fusion Network	RFM	Region-aware Fusion Module
AABTS-Net	Axial Attention Brain Tumor Segmentation Network	MCC	Modality-level Cross Connection	AFFM	Attentional Feature Fusion Module
MSFF	Multi-Scale Spatial Feature Fusion	DCFF	Dual Path Channel Feature Fusion	MAF-Net	Modality-Level Attention Fusion Network
DP	Dual Path	MAF	Multi-scale Attention Fusion	IDCM	Iterative Dilated Convolution Merging
mPMRI	Multi Parametric MRI	FFCM	Fast Fuzzy C Means	WT	Whole Tumor
TC	Tumor Core	ET	Enhance Tumor	MFD-Net	Modality Fusion Diffractive Network
GAM-Net	Gradient Assisted Multi-category Network	MSFR-Net	Multi-modality and Single-modality Feature Recalibration Network	DRM	Dual Recalibration Module
ViTBIS	Vision Transformer for Biomedical Image Segmentation	NMaFA	Nested Modalityaware Feature Aggregation	EMSViT	Efficient Multi-Scale Vision Transformer
MMCFormer	Missing Modality Compensation Transformer	TC-inception	Transformer-Convolution Inception mechanism	CAFGL	Cross-Attention Fusion with a Global and Local feature
SCCAF	Skip Connection with Cross-Attention Fusion	GAN	Generative Adversarial Network	AST	Axial Spatial Transformer
MLP	Multilayer Perceptron	VAE	Variational Autoencoder	CBAM	Convolution Block Attention Module
ESAB	Edge Spatial Attention Block	MFIB	Multi-Feature Inference Block	F2 Net	Flexible Fusion Network
CFM	Cross-modal Feature-enhanced Module	MCM	Multi-modal Collaboration Module	GSP	Ghost Spatial Pyramid
GSA	Ghost Self Attention	DRG	Dense Residual Ghost	TP	True Positive
FP	False Positive	FN	False Negative	TN	True Negative
DSC	Dice Similarity Coefficient	IoU	Intersection over Union	HD	Hausdorff distance
NLP	Natural Language Processing	MICCAI	Medical Imaging Computing and Computer-Aided Intervention Association	CE	Cross Entropy
BCE	Binary Cross Entropy	WCE	Weighted Cross Entropy	PWCE	Pixel Wise Cross Entropy
SHAP	SHapley Additive exPlanations	Lime	Local Interpretable Model-agnostic Explanations	XAI	Explainable AI

### 1.2 Related work

AI has demonstrated notable advancements in medical imaging, specifically in image processing and computer vision. AI models have emerged as a powerful tool for automating tasks like classifying, detecting, and segmenting tumor lesions. Thus, utilizing the capabilities of AI, these models improve the accuracy of segmentation results, consequently improving the quality of patient care. Current research contains comprehensive surveys that dive into cutting-edge advancements, particularly multi-modality MRI segmentation. Table 3 concisely summarizes previous studies conducted on the segmentation of brain tumors using multi-modality MRI with their essential features and weaknesses while briefly describing our proposed survey. The research conducted in (Wang et al., 2023b) provides a comprehensive overview of state-of-the-art (SOTA) vision transformers (ViT) employed in the segmentation of multi-modal brain MRI, along with the associated challenges and their potential future directions. However, it focuses more on statistical analysis of brain tumor segmentation.

**TABLE 3:** Summary of related survey articles.

Reference	Essential features	Weaknesses	Year
Wang et al. (2023b)	• Provide SOTA analysis of ViT for brain tumor segmentation • Include brain tumor databases	• It focuses more on the statistical analysis of the model	2023
Liu et al. (2023)	• Analysed DL-based multi-modality MRI brain tumor segmentation • Future trends were discussed	• Do not include a discussion on multi-modality brain MRI.	2023
Mohammed et al. (2023)	• Analysed machine learning, DL, and hybrid techniques for brain tumor segmentation using multi-modality MRI.	• Focus more on a general discussion of various techniques than architecture • Discussion on existing challenges and their possible future direction is neglected	2023
Ranjbarzadeh et al. (2023)	• Described machine learning and DL models • Overview of performance measures used in the segmentation	• Do not include the state-of-the-art vision transformers • Discussion on existing challenges and their possible future direction is neglected	2022
Ali et al. (2022)	• Discussed BraTS challenges from 2012 to 2018	• Do not discuss the problems in the BraTS challenges • The survey is more specific to the BraTS challenges than the architectural and performance improvements	2019
Proposed Survey	• Examine the prior research, which is based on CNN, transformer, and hybrid models, and cover their architecture in depth • Perform a thorough comparison of the multi-modal brain tumor segmentation model’s performance • Provide statistical analysis of recent research articles, widely utilized datasets, and evaluation metrics • Highlight open research challenges for brain tumor segmentation using multi-modal MRI images and suggest a possible future direction		

In (Liu et al., 2023), authors analyzed various DL methods for brain tumor segmentation. In (Mohammed et al., 2023), authors analyzed machine learning, DL, and hybrid techniques for brain tumor segmentation using multi-modality MRI. However, the discussion on challenges and their future direction was neglected. In (Ranjbarzadeh et al., 2023), the author analyzed the supervised and unsupervised DL models used in multi-modal brain tumor segmentation. However, they did not cover the main limitations and possible ways forward. In (Ali et al., 2022), the authors covered BraTS challenges from 2012 to 2020 but did not discuss the problems with BraTS challenges. and the survey is more specific to the BraTS challenges than the architectural and performance improvements. Our proposed review aims to conduct a comprehensive analysis and comparison of DL-based methods and their architectures for multi-modal MRI. Additionally, we will include statistical analyses of recent research articles, widely utilized datasets, evaluation measures, and a thorough comparison of segmentation performance. To address existing knowledge gaps and improve the reliability and efficiency of DL-based models for multi-modal brain tumor lesion segmentation using MRI, we will emphasize open research challenges and suggest potential future directions.

### 1.3 Contributions

This review predominantly focuses on using DL in multi-modal brain tumor MRI segmentation. Presently, DL demonstrates exceptional proficiency in this, exhibiting SOTA performance. In addition, we endeavor to examine the existing challenges and provide potential direction for future research from diverse perspectives. In summary, this review presents the subsequent significant contributions.

• We investigate several aspects of current DL-based methodologies employed for brain tumor segmentation. These aspects encompass the background, datasets utilized, models employed, and current progress trends in this field.

• We summarize the most recent CNN-based, transformer-based, and hybrid models to segment brain tumors. These models specifically focus on utilizing multi-modal MRI data and thoroughly comparing segmentation performance.

• Our study offers an extensive statistical analysis of recent research articles, widely utilized datasets, and evaluation metrics used in the multi-modal brain tumor segmentation.

• We highlight open research challenges for DL-based brain tumor segmentation using multi-modal MRI images and suggest a possible future direction, emphasizing extending the ability to enhance segmentation.

### 1.4 Organization of the paper

**FIGURE 2:**
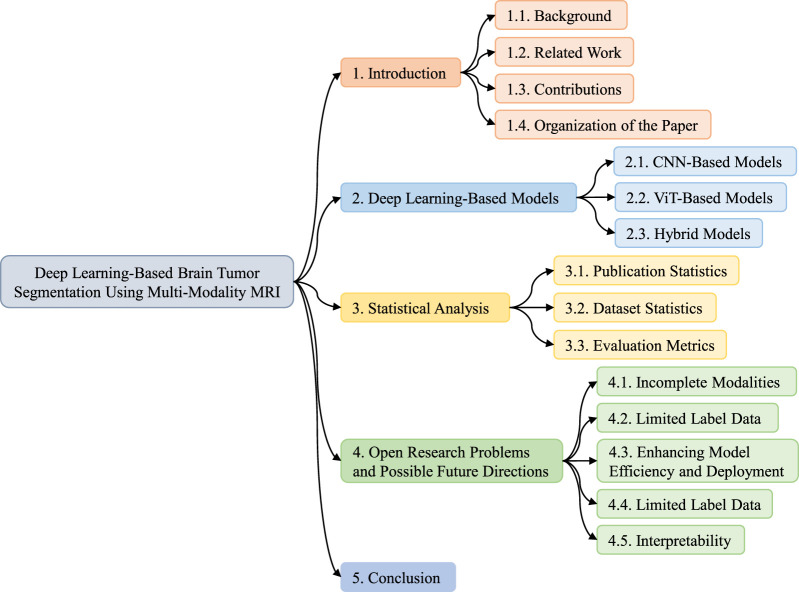
Organization of the DL-based brain tumor segmentation using multi-modality MRI survey.

This study is structured to provide a comprehensive understanding of multi-modal brain tumor segmentation. Each section highlights the various aspects involved in segmenting and evaluating brain tumors using multi-modal MRI, as shown in Figure 2. Section 1 provides an overview of this study. This section is divided into four subsections: background, related work, contributions, and organization of the paper. In Section 2, recent SOTA studies focusing on DL-based brain tumor segmentation using MRI are described. This section is divided into three subsections based on the model architecture: CNN, vision transformer, and hybrid models. Section 3 comprises a comprehensive statistical analysis and is divided into three subsections: publication statistics, datasets statistics, and evaluation metrics. Section 4 highlights some open research challenges in DL-based multi-modal MRI brain tumor segmentation and proposes possible future directions. Finally, Section 5 concludes the survey.

## 2 Deep learning-based multi-modality MRI brain tumor segmentation models

**FIGURE 3:**
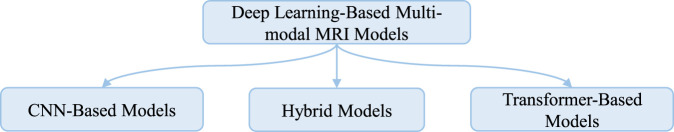
Classification of DL-based multi-modal MRI brain tumor segmentation models.

Medical image analysis has experienced an enormous revolution in recent years with the advent of powerful DL models. This paradigm change is most visible and important in multi-modal MRI brain tumor segmentation. The precise and efficient delineation of brain tumors is critical for clinical diagnosis, therapy planning, and ongoing patient monitoring (Philip et al., 2022). In response to this necessity, this analysis thoroughly examines improvements in DL-based models specifically designed for the challenging task of segmentation of brain tumors using MRI data. These models have demonstrated unparalleled success in extracting meaningful features from the diverse information encapsulated in MRI modalities by incorporating cutting-edge technologies such as CNNs, ViT models, and innovative hybrid architectures, combining both strengths. Figure 3 shows the classification of DL-based models according to the architecture and organization of this section.

The research highlights the technical complexities inherent in the models and underscores their novel influence on transforming the domain of medical imaging analysis. The ongoing purpose for improved accuracy, generalization, and interpretability urges scholars to continuously analyze and develop new systems and methodologies. The core purpose of this endeavor is to enhance the practicality of brain tumor segmentation models using MRI data, hence introducing an era of effectiveness in patient treatment. A detailed assessment of the DL-based models for multi-modal MRI brain tumor segmentation indicates a dynamic interaction of evolving architectures. Researchers are exploring this challenging domain with a desire for innovation, from the impressive power of CNNs to the transformational promise of vision transformer models. The use of CNNs, with their inherent capacity to acquire hierarchical features automatically, has prepared the path for ground-breaking advances.

The section on CNN-based models delves into the different architectural details, training methodologies, and data that explain their effectiveness. Concurrently, the introduction of vision transformer models marked a new era in image processing. Vision transformers provide a new viewpoint for feature extraction and fusion in multi-modal MRI data by relying on self-attention processes and the capacity to perceive global contextual information. The investigation of vision transformer-based models helps to reveal the distinct characteristics and exciting possibilities they bring ahead. Recognizing the combined effect and complementarities of both CNNs and vision transformers, the section on hybrid models delves into how these integrated architectures strive to strike an optimal balance between local and global information, aiming to push the boundaries of accuracy and robustness in brain tumor segmentation. The ongoing interaction of these diverse techniques and the dynamic growth of model architectures highlight the vibrant field of DL in multi-modal MRI brain tumor segmentation, offering a future where precision and clinical relevance merge to improve patient outcomes.

### 2.1 CNN-based models

CNNs have emerged as important tools in the fast-developing field of medical image processing, showing exceptional proficiency across a wide range of imaging applications (Singha et al., 2021). Within the domain of multi-modal MRI brain tumor segmentation, CNN-based models distinguish themselves through their exceptional performance and ongoing evolution. This section embarks on a detailed exploration, delving into the complexities of the various architectures and tactics used by CNN-based models. The exploration intends to highlight the complexity inherent in multi-modal MRI data, where the fusion of many imaging modalities brings distinct challenges. The present study provides nuanced insights into the CNNs for brain tumor segmentation, ranging from the typical effectiveness of 2D CNNs to the more advanced and volumetric capabilities of 3D variants.

One of the most important CNN-based architectures is UNet, designed for semantic segmentation tasks, particularly in medical image analysis (Ronneberger et al., 2015). It was introduced by Ronneberger et al., in 2015 and has since become widely used due to its effectiveness in producing accurate segmentation masks while efficiently handling limited training data. The UNet architecture consists of a contracting path, which captures context and reduces resolution, followed by an expanding path, which enables precise localization. Its unique feature is the skip connections that concatenate feature maps from the contracting path to the corresponding layers in the expanding path. These skip connections help preserve spatial information, allowing the model to produce detailed segmentations even for small structures in the input images. Despite its success, the original UNet architecture has certain limitations, such as struggles with handling class imbalance (Oktay et al., 2018) and difficulties in segmenting objects of varying sizes effectively (Zhou et al., 2018).

The authors in (Akbar et al., 2021) enhanced the UNet model for brain tumor segmentation by including attention, multiple atrous convolutions, and a residual route. This modified version is referred to as the Multiple Atrous Convolutions Attention Block (MAAB). The expansion part is included by extracting pyramid characteristics from each level and using them to generate the ultimate segmentation result. In (Syazwany et al., 2021), the authors proposed a multi-modal fusion network that incorporates a bi-directional feature pyramid network (MM-BiFPN). This network performs feature extraction from each modality using a separate encoder. The main objective is to use the intricate interactions across these modalities effectively. Furthermore, via the use of the bi-directional feature pyramid network (Bi-FPN) layer, they specifically concentrate on the combination of various modalities to examine the interrelationship between different modalities and the features at numerous scales.

The work in (Zhang et al., 2021) presented an innovative approach for segmenting brain tumors using a cross-modality deep feature learning framework. The fundamental concept is to extract valuable patterns to compensate for the limited amount of data available. The proposed framework for deep feature learning across different modalities comprises two distinct learning processes: the cross-modality feature transition (CMFT) process and the cross-modality feature fusion (CMFF) process. The CMFT process focuses on transferring knowledge between different modalities to learn comprehensive feature representations. On the other hand, the CMFF process aims to merge knowledge from various modalities to enhance the feature representations.

In (Fang et al., 2021), the proposed framework utilizes a hybrid fusion technique to combine data from different modalities. The authors also include a self-supervised learning method in this approach, and it relies on a fully CNN. Initially, they provide an architecture with multiple inputs that acquire distinct characteristics from multi-modal data. The model outperforms single-modal multi-channel networks by offering an improved feature extractor for segmentation tasks. This feature extractor effectively captures cross-modal information from multi-modal input. Furthermore, they provide a novel method for combining features, which they refer to as hybrid attentional fusion. This technique allows the acquisition of the hybrid representation of various characteristics and the collection of correlation information via an attention mechanism. Contrary to commonly used techniques like feature map concatenation, this approach has a complementary nature of multi-modal data, resulting in remarkable progress in the segmentation outcomes of certain areas.

The study in (Zhou et al., 2021) introduced an innovative neural network for segmenting brain tumors when one or more modalities are absent. The network has three sub-networks: a feature-enhanced generator (FeG), a correlation constraint (CC), and segmentation. The FeG employs the existing modalities to create a three-dimensional image that enhances the features and represents the missing modality. The CC block leverages the multi-source correlation and restricts the generator to produce a modality enriched with features consistent with the existing modalities. The segmentation network utilizes a U-Net architecture with multiple encoders to perform brain tumor segmentation accurately. In (Wang et al., 2021b), the authors developed an innovative end-to-end modality-pairing learning approach for segmenting brain tumors. The goal of paralleled branches is to use distinct modality traits, while a network of layer connections is employed to collect intricate interactions and ample information across modalities. In addition, they use consistency loss to reduce the variability in predictions across two branches. Finally, they use an average ensemble of different models together with various post-processing approaches to obtain the ultimate outcomes.

The authors in (Ding et al., 2021a) introduced a Region-aware Fusion Network (RFNet) that can intelligently and efficiently use various combinations of multi-modal data for tumor segmentation. The researchers have developed a Region-aware Fusion Module (RFM) in RFNet to combine features from multiple image modalities based on specific brain tumor locations since different modalities are sensitive to different regions. RFNet utilizes RFM to intelligently segment tumor areas from a limited collection of multi-modal images by efficiently combining modal data. In addition, they also create a segmentation-based regularizer to address the issue of inadequate and imbalanced training in RFNet due to missing multi-modal data. More precisely, in addition to acquiring segmentation outcomes from combined modal features, they also segment each imaging modality separately using the associated encoded features. By using this approach, every modal encoder is compelled to acquire distinguishing characteristics, hence enhancing the capacity of the combined features to represent information.

The CNN model in (Tong and Wang, 2023) has a distinctive architecture with two prominent characteristics. The feature extraction block has three pathways to extract full feature information from the multi-modality input. Each path is responsible for extracting features from mono-modality, paired-modality, and cross-modality data. Furthermore, it possesses a distinct tri-sectioned categorization system to differentiate pixels belonging to three intra-tumoral groups from the surroundings. The branches are trained individually to ensure that the updating process is applied to the parameters precisely using the matching annotations of the target tumor locations. In (Zhao L. et al., 2022), a multi-modality feature fusion network called MM-UNet was developed. This network utilizes a structure with several encoders and a single decoder to perform brain tumor segmentation. Within the proposed network, individual encoders autonomously extract low-level characteristics from their respective imaging modalities, while the hybrid attention block enhances the features. The decoder utilizes skip connections to include high-level semantic information and provide accurate pixel-level segmentation results.

The researchers in (Tian et al., 2022) devised an axial attention brain tumor segmentation network (AABTS-Net) to automatically delineate tumor sub-regions using multi-modality MRIs. The axial attention mechanism aids in the acquisition of deeper semantic information, facilitating models by offering both local and global information while reducing computing complexity. The use of the deep supervision mechanism serves the purpose of preventing the occurrence of vanishing gradients and providing guidance to the AABTS-Net to provide enhanced feature representations. The authors in (Zhou et al., 2023) introduced a modality-level cross-connection (MCC) network, which is a 3D UNet based on several encoders designed for brain tumor segmentation. The MCC network leverages beneficial information between the different modalities. Additionally, to improve its ability to learn features, the researchers introduced the attentional feature fusion module (AFFM). This module combines many modalities and extracts valuable feature representations for segmentation. The AFFM comprises two main elements: the multi-scale spatial feature fusion (MSFF) block and the dual-path channel feature fusion (DCFF) block. Their objective is to acquire multi-scale spatial and channel-wise feature information to enhance the accuracy of segmentation.

The authors in (Liu et al., 2022) proposed a multi-modal image fusion approach that combines pixel- and feature-level fusion to improve the effectiveness and precision of brain tumor segmentation. The goal is to enhance the exploitation of multi-modal information. They introduced a convolutional network called PIF-Net for 3D MR image fusion at the pixel level, enhancing the segmentation model’s input modalities. The integration of numerous source modalities might increase the correlation between various forms of disease information, resulting in an amplification of modality effects. At the feature level, attention-based modality selection feature fusion is designed to improve multi-modal features by addressing the variations among different modalities for a certain segmentation objective. In the (Huang et al., 2022), the authors introduced a modality-level attention fusion network (MAF-Net), which uses patchwise contrastive learning to extract latent features from several modalities. Additionally, attention weights are dynamically assigned to fuse the distinct modalities uniquely.

The work in (Chang et al., 2023) introduced a 3D segmentation model called DPAFNet. This model is based on integrating a dual-path (DP) module and a multi-scale attention fusion (MAF) module. The DPAFNet utilizes DP convolution to expand capacity and incorporates residual connections to prevent deterioration. An attention fusion module combines global and local information at the channel level. This module fuses feature maps of various sizes to provide enriched features with enhanced semantic information. This prioritizes the comprehensive examination of tiny cancers. In addition, the 3D iterative dilated convolution merging (IDCM) module also enhances the receptive field and contextual awareness.

A novel approach is presented in (Sahoo et al., 2023), which combines the Inception V2 network with 16 newly developed layered segmentation nets to create a hybrid deep neural network. The network undergoes testing using the BraTs 2020 and BraTs 2017 multi-parametric MRI (mPMRI) datasets to identify the whole tumor. To recognize the tumor core (TC) and the edema, the fast fuzzy C-means (FFCM) algorithm is used. In (Hou et al., 2023), the authors proposed a modality fusion diffractive network (MFD-Net) for accurately and automatically segmenting brain tumors. The MFD-Net consists of diffractive blocks and modality feature extractors. The diffractive block, constructed using Fraunhofer’s single-slit diffraction principle, highlights nearby feature points with high confidence while reducing the prominence of low-quality or isolated feature points. This improves the interconnectedness of the features. Adopting a global passive reception mode resolves the problem of fixed receptive fields. The self-supervised technique efficiently exploits the inherent generalization information of each modality to extract modality features. This allows the main segmentation branch to prioritize the fusion of multi-modal feature information.

The work in (Çetiner and Metlek, 2023) introduced DenseUNet+, a novel DL method for achieving precise segmentation of multi-modal images. The DenseUNet + model included data from four distinct modalities in dense block structures. Subsequently, the data underwent linear operations followed by the concatenate operation. The findings acquired using this method were transmitted to the decoder layer. In (Wang et al., 2023), the authors introduced a novel segmentation network called a gradient-assisted multi-category network (GAM-Net). GAM-net consists of three components: a double convolution encoder, a gradient extraction branch, and a gradient-driven decoder. A double convolution encoder extracts detailed features from MRI images; a gradient extraction branch generates gradient features to aid in area segmentation, and a gradient-driven decoder effectively combines contour information and encoding features.

The researchers in (Li et al., 2023a) introduced multi-modality and single-modality feature recalibration network (MSFR-Net). Distinct pathways handle the flow of multi-modality and single-modality information. The multi-modality network captures the correlations relating to different modalities and various tumor sub-components. A single-modality network is trained to understand the connection between a single modality and its closely related tumor subcomponents. Subsequently, a dual recalibration module (DRM) is devised to establish a connection between the parallel single-modality network and the multi-modality network at various phases. The purpose of the DRM is to integrate the two kinds of features into a single feature space.

In this subsection, the advancement of CNN-based brain tumor segmentation models using multi-modal MRI signifies notable progress in medical image analysis. Since its first implementation to the current advanced 3D versions, CNNs have been crucial in improving the precision of segmentation. However, their inherent limitation in capturing global characteristics has facilitated the development of later advancements. As we recognize the accomplishments and continued difficulties in this field, the persistent effort to improve CNN designs and methodologies highlights their ongoing importance in accurately and therapeutically useful brain tumor segmentation. Finally, the reviewed studies that used the CNN-based model are summarized in Table 4.

**TABLE 4:** CNN-based models for multi-modal MRI brain tumor segmentation.

Segmentation models	Dataset	Experimental parameters		Segmentation performance					Ref.
		Optimizer	Loss function	WT		TC		ET	
UNet with Multiple Atrous convolutions Attention Block (MAAB)	BraTS 2021	Adam	dice	DSC = 0.884 HD = 10.70		DSC = 0.829 HD = 23.01		DSC = 0.817 HD = 19.70	Akbar et al. (2021)
Multi-Modality Bi-directional Feature Pyramid Network (MM-BiFPN)	BraTS 2018	Adam	CE	DSC = 0.811		DSC = 0.777		DSC = 0.735	Syazwany et al. (2021)
	BraTS 2020			DSC = 0.836		DSC = 0.815		DSC = 0.779	
Cross Modality Deep Feature Learning	BraTS 2017	Adam	-	DSC = 0.898 HD = 5.155		DSC = 0.823 HD = 6.999		DSC = 0.762 HD = 3.170	Zhang et al. (2021)
	BraTS 2018			DSC = 0.903 HD = 4.998		DSC = 0.836 HD = 6.639		DSC = 0.791 HD = 3.992	
Self-Supervised Learning Model	BraTS 2019	Adam	CE and dice	DSC = 0.927 HD = 2.446		DSC = 0.895 HD = 1.783		DSC = 0.835 HD = 1.623	Fang et al. (2021)
Feature Enhance Generation and Multi-Modality Fusion Based Network	BraTS 2018	Nadam	dice	DSC = 0.866		DSC = 0.858		DSC = 0.769	Zhou et al. (2021)
Madality-Paring Learning	BraTS 2020	SGD	CE and dice	DSC = 0.891 HD = 6.24		DSC = 0.842 HD = 19.54		DSC = 0.816 HD = 17.79	Wang et al., 2021b
Region-aware Fusion Network (RFNet))	BraTS 2015	Adam	WCE and dice	DSC = 0.861		DSC = 0.719		DSC = 0.589	Ding et al. (2021a)
	BraTS 2018			DSC = 0.857		DSC = 0.765		DSC = 0.571	
	BraTS 2020			DSC = 0.869		DSC = 0.782		DSC = 0.615	
CNN Model with Feature Extraction (FE) Block	BraTS 2018	-	-	DSC = 0.886		DSC = 0.801		DSC = 0.787	Tong and Wang (2023)
	BraTS 2019			DSC = 0.885		DSC = 0.776		DSC = 0.751	
Multi-Modality Fusion network (MM-UNet)	BraTS 2020	Adam	dice and focal	DSC = 0.850 HD = 8.243		DSC = 0.765 HD = 10.76		DSC = 0.762 HD = 6.389	Zhao et al. (2022a)
Axial Attention CNN for Brain Tumor Segmentation (AABTS-Net)	BraTS 2019	Adam	BCE and dice	DSC = 0.911 HD = 3.988		DSC = 0.838 HD = 6.028		DSC = 0.777 HD = 3.246	Tian et al. (2022)
	BraTS 2021			DSC = 0.922 HD = 3.996		DSC = 0.861 HD = 11.18		DSC = 0.830 HD = 17.73	
Modality-Level Cross Connection (MCC)	BraTS 2018	Nadam	dice	DSC = 0.865 HD = 4.60		DSC = 0.870 HD = 3.60		DSC = 0.794 HD = 2.50	Zhou et al. (2023)
Pixel level and Feature level Image Fusion Network	BraTS 2019	Adam	BCE and dice	DSC = 0.894 HD = 5.349		DSC = 0.814 HD = 10.89		DSC = 0.771 HD = 5.855	Liu et al. (2022b)
	BraTS 2020			DSC = 0.895 HD = 5.312		DSC = 0.817 HD = 9.429		DSC = 0.775 HD = 4.472	
Modality-level Attention Fusion Network (MAF-Net)	BraTS 2020	Adam	CE	DSC = 0.880		DSC = 0.679		DSC = 0.418	Huang et al. (2022a)
Pixel-level and Feature-level Image Fusion Network for Brain Tumor Segmentation	BraTS 2018	Adam	CE	DSC = 0.900 HD = 6.51		DSC = 0.839 HD = 5.71		DSC = 0.795 HD = 2.92	Chang et al. (2023)
	BraTS 2019			DSC = 0.890 HD = 8.53		DSC = 0.812 HD = 7.43		DSC = 0.782 HD = 3.82	
	BraTS 2020			DSC = 0.894		DSC = 0.832		DSC = 0.781	
Improve DNN with fast Fuzzy C-Means (FFCM)	BraTS 2017	-	-	DSC = 0.891		DSC = 0.847		DSC = 0.865	Sahoo et al. (2023)
	BraTS 2020			DSC = 0.904		DSC = 0.858		DSC = 0.865	
Modality Fusion Diffractive Network (MFD-Net)	BraTS 2018		BCE and dice	DSC = 0.908 HD = 5.986		DSC = 0.856 HD = 6.995		DSC = 0.767 HD = 3.409	Hou et al. (2023)
	BraTS 2019			SGD		DSC = 0.857 HD = 5.83		DSC = 0.767 HD = 3.41	
	BraTS 2021			DSC = 0.927 HD = 3.51		DSC = 0.887 HD = 5.77		DSC = 0.854 HD = 13.98	
DenseUNet + Model	FeTS 2021	Adam	dice	DSC = 0.883		DSC = 0.862		DSC = 0.865	Çetiner and Metlek (2023)
	BraTS 2021			DSC = 0.958		DSC = 0.955		DSC = 0.937	
Gradient Assisted Multi-Category (GAM-Net) Network	BraTS 2020	Adam	dice	DSC = 0.899 HD = 5.076		DSC = 0.840 HD = 5.096		DSC = 0.758 HD = 5.296	Wang et al. (2023c)
Multi-Modality and Single-Modality Feature Recalibration Network (MSFR-Net)	BraTS 2015	Adam	CE and dice	DSC = 0.860		DSC = 0.740		DSC = 0.650	Li et al. (2023a)
	BraTS 2018			DSC = 0.909 HD = 4.24		DSC = 0.858 HD = 6.72		DSC = 0.807 HD = 2.73	

### 2.2 Vision transformer-based models

The introduction of ViT models represents an architectural change in image analysis, demonstrating effectiveness across several domains. Vision transformers provide a unique viewpoint for feature extraction and fusion by depending on self-attention mechanisms and acquiring global contextual information. This section examines the use of vision transformer models in brain tumor segmentation using multi-modal MRI, shedding light on their potential to improve segmentation accuracy and resilience in the context of multi-modal MRI data. In (Sagar et al., 2021a), the authors proposed a ViT for biomedical image segmentation (ViTBIS) model. The model divides input feature maps into three parts using 1 × 1, 3 × 3, and 5 × 5 convolutions in the encoder and decoder. The concatenation operator merges features before feeding them to three transformer blocks with attention mechanisms. Skip connections link encoder and decoder transformer blocks. Before linearly projecting the output segmentation map, decoders employ transformer blocks and a multi-scale architecture.

In (Pinaya et al., 2022), authors use vector quantized variational autoencoders’ latent representation and an ensemble of autoregressive transformers to identify and segment unsupervised anomalies based on brain imaging data deviation at a low computing cost. They achieve improved image- and pixel-wise anomaly detection without post-processing. These findings highlight transformers’ potential in this most difficult imaging job. The work in (Peiris et al., 2022a) presents a novel Transformer architecture designed specifically for volumetric segmentation. This is challenging as it effectively captures and incorporates local and global spatial inputs while conserving information across volume axes. The proposed design’s encoder leverages a self-attention mechanism to simultaneously encode local and global cues. Meanwhile, the decoder utilizes a parallel formulation of self and cross-attention to effectively capture intricate features for boundary refinement. The proposed model is computationally efficient and exhibits competitive and promising outcomes when applied to the BraTS Task.

The authors of (Peiris et al., 2022b) introduced a model that constructs a U-shaped Volumetric Transformer (CR-Swin2-VT) using two well-known window-based attention mechanisms: the Cross-shaped window attention-based Swin Transformer block and the Shifted window attention-based Swin Transformer block. The CR-Swin2-VT model employs a parallel configuration of Swin Transformer blocks and CSWin Transformer blocks to capture voxel information on the encoder side. However, on the decoder side, only Swin Transformer blocks are utilized. In (Xing et al., 2022), authors presented a Nested Modality Aware Transformer (NestedFormer) that investigates the inter- and intra-modality relationships. They implemented modality-sensitive gating (MSG) at lower scales to facilitate more efficient skip connections and conduct nested multi-modal fusion for high-level representations of distinct modalities, utilizing a transformer-based multi-encoder and single-decoder architecture. Their proposed Nested Modality-aware Feature Aggregation (NMaFA) module provides the basis for performing multi-modal fusion. This module utilizes a cross-modality attention transformer to supplement critical contextual information among modalities and a tri-orientated spatial attention transformer to enhance long-term dependencies within individual modalities.

The authors in (Sagar et al., 2021b) proposed an efficient multi-scale ViT (EMSViT) that divides the input into three parts with various convolution sizes. Feature maps are merged before being fed into the three transformer blocks. In the decoder, transformer blocks and a multi-scale architecture are used to facilitate the linear projection of the input, resulting in the generation of the output segmentation map. In (Liu et al., 2022c), authors introduced a self-attention-based fusion block (SFusion). The proposed block automatically fuses available modalities without zero-padding missing ones. To produce latent multi-modal correlations, project feature representations from the upstream processing model as tokens and feed them into the self-attention module. The self-attention module generates latent multi-modal correlations from upstream processing model feature representations projected as tokens. A modal attention technique builds a common representation for the downstream decision model. The proposed SFusion integrates readily into multi-modal analytic networks, and they use SFusion on several backbone networks to segment brain tumors.

The authors in (Karimijafarbigloo et al., 2023) proposed the missing modality compensation transformer (MMCFormer) to handle missing information. They utilized 3D-efficient transformer blocks and co-training to efficiently train a missing modality network. MMCFormer uses global contextual agreement modules in each encoder scale to maintain feature consistency across many scales. Further, they used auxiliary tokens at the bottleneck stage to depict the interaction between full and missing-modality channels to transmit modality-specific concepts. Moreover, they included feature consistency losses to minimize network prediction domain gaps and enhance reliability for missing modality paths.

In this subsection, we investigate the ViT for brain tumor segmentation using multi-modal MRI. ViT overcomes a significant drawback of CNNs by using self-attention processes to gather global contextual information effectively. As we acknowledge the fundamental change introduced by transformers, their incorporation into the rapidly evolving field of medical image processing has significant potential to improve accuracy and reliability in brain tumor segmentation tasks. Finally, the reviewed studies that used the transformer model are summarized in Table 5.

**TABLE 5:** Vision transformer-based models for multi-modal brain tumor segmentation.

Segmentation models	Dataset	Experimental parameters		Segmentation performance			Year	Ref.
		Optimizer	Loss function	WT	TC	ET		
ViT for biomedical image segmentation (ViTBIS)	BraTS 2019	Adam	BCE and dice	DSC = 0.903 HD = 5.621	DSC = 0.822 HD = 7.129	DSC = 0.792 HD = 3.71	2021	Sagar et al. (2021a)
Vector Quantised Variational Autoencoder with an Ensemble of Autoregressive Transformer	BraTS 2018	-	-	Avg. DSC = 0.537 Avg. AUPRC = 0.555			2022	Pinaya et al. (2022)
Volumetric transformer UNet (VT UNet)	MSD	AdamW	-	DSC = 0.919 HD = 3.51	DSC = 0.872 HD = 4.10	DSC = 0.822 HD = 2.68	2022	Peiris et al. (2022a)
CR-Swin2-VT	FeTS	Adam	CE, dice and VAT	DSC = 0.914 HD = 3.93	DSC = 0.854 HD = 11.19	DSC = 0.817 HD = 14.81	2022	Peiris et al. (2022b)
Nested Modality-Aware Transformer (NestedFormer)	BraTS 2020	AdamW	CE and soft dice	DSC = 0.920 HD = 4.567	DSC = 0.864 HD = 5.316	DSC = 0.800 HD = 5.269	2022	Xing et al. (2022)
Efficient multi-scale ViT (EMSViT)	BraTS 2019	Adam	BCE and dice	DSC = 0.903 HD = 5.621	DSC = 0.822 HD = 7.129	DSC = 0.792 HD = 3.71	2022	Sagar et al. (2021b)
Self-attention based N-to-One multi-modal fusion (SFusion)	BraTS 2020	Adam	CE	DSC = 0.889	DSC = 0.822	DSC = 0.738	2023	Liu et al. (2022c)
Missing modality compensation transformer (MMCFormer)	BraTS 2018	Adam	dice	DSC = 0.890	DSC = 0.874	DSC = 0.801	2023	Karimijafarbigloo et al. (2023)

### 2.3 Hybrid models

Hybrid models combine the benefits of both CNN and transformer. Many studies prefer to combine these two to improve the model’s performance. CNNs struggle to capture global feature relations, affecting segmentation accuracy (Li et al., 2024). Thus, a Transformer network is developed, which can capture global information but not local details and requires pre-training on big datasets (Zhang et al., 2023). Therefore, the hybrid model overcomes the limitations by combining their strengths and aims to strike a superior balance between local and global information. The authors in (Wang et al., 2021) utilize a Transformer in 3D CNN for the first time and propose a TransBTS. To obtain the local 3D context information, the encoder initially extracts the volumetric spatial feature maps using 3D CNN. In the meantime, the tokens from the feature maps are precisely transformed and input into a Transformer to model global features. To predict the detailed segmentation map, the decoder employs progressive upsampling and utilizes the features embedded by the Transformer.

In (Li et al., 2021), the authors introduced Segtran, a transformer-based segmentation technique with infinite effective receptive fields at high feature resolutions. Segtran uses a unique squeeze-and-expansion transformer to regularize self-attention and learn diverse representations. Additionally, they introduced a transformer positional encoding method with a continuous inductive bias for images. The authors in (Jun et al., 2021) introduced a medical transformer, a transfer learning architecture that models 3D volumetric images as 2D image slices. For improved 3D-form representation of spatial relations, they utilized a multi-view technique that integrates information from the three planes of 3D volume and offers parameter-efficient training. They use a large-scale normal, healthy brain MRI dataset to pre-train a source model for masked encoding vector prediction, which may be used for numerous purposes.

The work in (Liang et al., 2022a) introduced a TransConver, a U-shaped segmentation network that utilizes convolution and transformer to provide automated and precise brain tumor segmentation in MRI images. In contrast to the transformer and convolution models that have been previously proposed, they have introduced a parallel module called transformer-convolution inception (TC-inception). This module utilizes convolution blocks to extract local information and transformer blocks to extract global information. These two types of information are integrated through a cross-attention fusion with a global and local feature (CAFGL) mechanism. The skip connection with cross-attention fusion (SCCAF) method is an enhanced structure that may mitigate the semantic disparities between encoder and decoder features, resulting in improved feature fusion.

In (Zhang et al., 2022a), the authors introduced a new multi-modal medical transformer (mmFormer) for incomplete multi-modal learning. It consists of three main parts: a hybrid modality-specific encoder that models both local and global contexts in every modality; An inter-modal transformer is designed to construct and synchronize long-range correlations among modalities to identify modality-invariant features that correspond to the global semantics of the tumor region; and a decoder that generates robust segmentation by progressive up-sampling and fusion with the modality-invariant features. Additionally, to make the model even more resistant to incomplete modalities, auxiliary regularizers are included in the encoder and decoder.

The authors in (Chen and Wang, 2022) introduced TSEUnet, a 3D nnUNet-based network. This network uses a parallel interactive transformer module in the encoder to extract local features and global contexts effectively. The decoder additionally uses SE-Attention to increase brain tumor segmentation and provide useful information. The authors in (Wang et al., 2022) designed a hybrid encoder-decoder that included lightweight convolution modules as well as an axial-spatial transformer (AST) module in the encoder. They intergrade axial and spatial attention in the AST module to capture better multi-view and multi-scale characteristics to learn long-range relationships, while convolution operations extract local dependencies and rich local characteristics.

To simplify the process of segmentation, the authors of (Liu et al., 2022) take advantage of a 2D backbone for segmenting a 3D brain tumor (Transition Net). To segment 3D brain tumor images, they make use of the Swin transformer as the encoder, in conjunction with a decoder that is produced by the process of 3D convolution. To address the issue of cross-domain variation, they developed the components known as the transition head to turn the input data into feature maps that are acceptable for Swin Transformer and the transition decoder to convert the multi-scale feature maps that were recovered by the backbone. After a series of stages, these maps are fused with the features sampled on CNN to obtain the final segmentation results.

In (Li et al., 2022), the authors aimed to use the Transformer model in a 3D CNN to segment 3D medical image volumes. They introduced a new model called TransBTSV2, built upon an encoder-decoder architecture. The proposed TransBTSV2 is not just restricted to brain tumors but emphasizes the broader medical image segmentation domain. It offers a more robust and efficient 3D foundation for the volumetric segmentation of medical images. TransBTSV2 is a hybrid CNN-transformer architecture that can accurately segment medical images without the need for pre-training. It integrates the strong permanent bias of CNNs with the excellent global context modeling capacity of transformers. By proposing a new approach to restructure the internal structure of the transformer block and introducing the deformable bottleneck module to capture shape-aware local information, they have produced a highly efficient architecture with higher performance.

The work in (Huang et al., 2022) introduced a generative adversarial network (GAN) based on transformers. To optimize the segmentation process, the network integrates the “generative adversarial” and “transformer” concepts. The generator network segments multi-modal MRI brain tumors using a transformer with the Resnet module in 3D CNN. The transformer and Resnet block efficiently capture local and global features, thereby facilitating the progressive upsampling of embedded features to generate full-resolution predicted maps. In (Liang et al., 2022b), the authors introduce an effective transformer-based model that incorporates a 3D parallel shifted window-based transformer module (3D PSwinBTS) to capture long-range contextual information. Additionally, to achieve efficient semantic modeling, they make use of semantic supervision to incorporate eight semantic priors into the encoder of the 3D PSwinBTS model.

In (Jia et al., 2021), the authors proposed a combined CNN-transformer model called BiTr-UNet. It contains the main characteristics and backbone of TransBTS. They validated their model on the BraTS 2021 datasets and achieved good performance. The authors in (Dobko et al., 2021) modified the original TransBTS by adding more CNN layers, squeeze-and-excitation (SE) blocks, and trainable multilayer perceptron (MLP) embeddings instead of positional encoding in the transformer block. This modification enables the transformer to be adjusted to accommodate inputs of any size while performing inference. In addition, they chose to integrate our improved TransBTS into the nnU-Net framework by making architectural modifications to the nnUNet model according to our custom model.

The authors in (Pham et al., 2022) introduced a novel model called SegTransVAE, which utilizes an encoder-decoder design, including a transformer and a variational autoencoder (VAE) in the model. SegTransVAE is a multitask learning model that can simultaneously achieve brain tumor segmentation and image reconstruction. In (Yang et al., 2021), the authors proposed a convolution-and-transformer network (COTRNet) to accurately gather global information, along with the implementation of a topology-aware (TA) loss to restrict the learning process to topological information. In addition, they use transfer learning by using pre-trained parameters from ImageNet and implement deep supervision by including multi-level predictions to enhance segmentation performance.

In (Futrega et al., 2021), the authors introduced a segmentation model called Swin UNEt TRansformer (Swin UNETR). The objective of 3D brain tumor semantic segmentation is transformed into a prediction problem where multi-modal input data is converted into a one-dimensional sequence of embeddings. This series is then fed into a hierarchical Swin transformer, which serves as the encoder. The Swin transformer encoder employs shifted windows to compute self-attention and extract features at five distinct resolutions. The authors in (Wang et al., 2022) introduced a Trans-NUNet model, they used a convolution block attention module (CBAM) in the model to improve the performance of each model while dealing with images of varying sizes throughout the stage. The CBAM models provide rapid identification of the region of interest within the feature map by the whole network, followed by a thorough analysis of that specific area.

The authors in (Hu et al., 2023) proposed a novel combination of R-Transformer and U-Net, an efficient R-Transformer with dual encoders (ERTN). To capture global information and complicated semantic characteristics, ERTN builds a feature branch and a patch branch. To achieve accurate localization, the decoder augments low- and high-resolution CNN data with up-sampled features produced by the feature branch and patch branch. Finally, ERTN uses the Transformer’s ranking attention mechanism (RTransformer), assisting the model in focusing on relevant data for enhanced training efficiency and decreased computing cost.

In (Zhu et al., 2023a), the authors proposed a model that fuses deep semantics with edge information. Semantic segmentation, edge detection, and feature fusion are the primary components of the proposed model. This module’s semantic segmentation makes use of the Swin Transformer for feature extraction and introduces a shifting patch tokenization technique for enhanced training. A CNN-based edge detection module is introduced, together with an edge spatial attention block (ESAB) for feature improvement. They developed a graph convolution-based multi-feature inference block (MFIB) to conduct feature reasoning and information dissemination to achieve successful feature fusion in the feature fusion module, which is responsible for merging the derived semantic and edge features.

The study in (Gao et al., 2023) incorporates transformer layers into a U-shaped design’s encoder and decoder using a deep mutual learning method. Due to the inherent complementarity between shallow features and deep features in a layer, where shallow features encompass plentiful spatial details but lack semantic information, conversely, the feature map of the shallowest layer is employed to guide the feature map of the deeper layers. This approach ensures that the deeper layers, which retain more edge information, guide the accuracy of sub-region segmentation. Employing the most profound classification logits to oversee the less profound logits to preserve a greater amount of semantic information for the differentiation of tumor sub-regions. Moreover, the shallow feature map and the deep logit mutually supervise each other, leading to an improvement in the overall accuracy of tumor segmentation.

The researchers in (Yang et al., 2023) introduced a flexible fusion network (F2 Net) for the segmentation of brain tumors. The F2 Net is built around an encoder-decoder structure, including two Transformer-based streams for feature learning and a cross-modal shared learning network to extract distinct and common feature representations. To efficiently incorporate information from multiple types of data, they suggested the use of a cross-modal feature-enhanced module (CFM) and a multi-modal collaboration module (MCM). The CFM is designed to combine features from different modalities in a shared learning network, while the MCM integrates features from encoders into a shared decoder.

The authors in (Lu et al., 2023) introduced a new 3D multi-scale Ghost CNN with an additional MetaFormer decoding path (GMetaNet). Efficient semantic information extraction was carried out Through the integration of CNN’s localized modeling and the Transformer’s capability for long-range representation. Three new modules are introduced, notably the lightweight Ghost spatial pyramid (GSP) module, the Ghost self-attention (GSA) module, and the dense residual Ghost (DRG) module, which are built upon the existing Ghost module. Furthermore, the GSP module efficiently acquires knowledge about various receptive fields to enhance the multiscale representation while reducing computational expenses. The GSA module allows the model to capture long-range relationships effectively. The DRG module, functioning as a local decoder, enhances information and prevents deterioration. Furthermore, a comprehensive decoder incorporating MetaFormer has been developed to combine local and global information successfully. Ultimately, the technique of deep supervision combines three outputs and enhances the rate at which the system reaches convergence.

To summarize, this section examines the latest research in brain tumor segmentation techniques, specifically focusing on the use of multi-modal MRI data. The field has seen a significant movement in segmentation methodologies, moving from the original use of CNNs to the introduction of transformers, and finally to the development of hybrid models. This transition has resulted in more comprehensive and effective segmentation techniques. The use of transformers, which excel at collecting global characteristics, complements the localized capabilities of CNNs in a mutually beneficial way. The ongoing development of multi-modal MRI brain tumor segmentation is driven by the junction of CNNs, transformers, and hybrid architectures, as we seek to achieve the most effective solutions. Finally, the reviewed studies that used the hybrid model are summarized in Table 6.

**TABLE 6:** Hybrid transformer models for multi-modal MRI brain tumor segmentation.

Segmentation models	Dataset	Experimental parameters		Segmentation performance					Year	Ref.
		Optimizer	Loss function	WT	TC			ET		
Multi-modal brain tumor segmentation using transformer (TransBTS)	BraTS 2019	Adam	dice	DSC = 0.900 HD = 5.644	DSC = 0.819 HD = 6.049			DSC = 0.789 HD = 3.736	2021	Wang et al., 2021a		
	BraTS 2020			DSC = 0.901 HD = 4.964	DSC = 0.817 HD = 9.769			DSC = 0.787 HD = 17.947				
Segmentation model based on squeeze and expansion transformer (SegTran)	BraTS 2019	AdamW	PWCE and dice	DSC = 0.895	DSC = 0.817			DSC = 0.740	2021	Li et al. (2021)		
Medical transformer	BraTS 2019	Adam	triplet	DSC = 0.873	DSC = 0.697			DSC = 0.588	2021	Jun et al. (2021)		
Convolution and transformer-based segmentation model (TransConver)	BraTS 2018	Adam	CE and dice	DSC = 0.859 HD = 2.587	DSC = 0.838 HD = 1.607			DSC = 0.789 HD = 2.692	2022	Liang et al. (2022a)		
	BraTS 2019			DSC = 0.859 HD = 2.587	DSC = 0.838 HD = 1.607			DSC = 0.789 HD = 2.692				
Multi-modal medical transformer (mmFormer)	BraTS 2018	Adam	dice	DSC = 0.896	DSC = 0.858			DSC = 0.776	2022	Zhang et al. (2022a)		
Transformer and SE-Attention (TSEUNet)	BraTS 2018	SGD	CE and dice	DSC = 0.911	DSC = 0.873			DSC = 0.824	2022	Chen and Wang (2022)		
Axial-spatial transformer network (AST-Net)	BraTS 2018	Adam	dice	DSC = 0.905 HD = 5.950	DSC = 0.850 HD = 9.200			DSC = 0.795 HD = 2.980	2022	Wang et al., 2022b		
	BraTS 2019			DSC = 0.899 HD = 5.49	DSC = 0.843 HD = 6.32			DSC = 0.786 HD = 2.90				
	BraTS 2020			DSC = 0.904 HD = 6.05	DSC = 0.842 HD = 6.12			DSC = 0.778 HD = 30.83				
2D backbone to segment 3D brain tumor (Transition Net)	BraTS 2019	AdamW	weighted region	DSC = 0.913 HD = 20.15	DSC = 0.845 HD = 12.21			DSC = 0.749 HD = 10.09	2022	Liu et al. (2022d)		
TransBTSV2	BraTS 2019	Adam	softmax dice	DSC = 0.904 HD = 5.432	DSC = 0.849 HD = 5.473			DSC = 0.802 HD = 3.696	2022	Li et al. (2022)		
	BraTS 2020			DSC = 0.904 HD = 5.432	DSC = 0.849 HD = 5.473			DSC = 0.802 HD = 3.696				
Generative adversarial network (GAN) based on transformers	BraTS 2018	Adam	dice	DSC = 0.902 HD = 5.418	DSC = 0.809 HD = 9.405			DSC = 0.769 HD = 5.712	2022	Huang et al. (2022b)		
	BraTS 2020			DSC = 0.903 HD = 4.909	DSC = 0.815 HD = 7.494			DSC = 0.708 HD = 37.579				
3D parallel shifted windows for brain tumor segmentation (3D PSwinBTS)	BraTS 2020	Adam	CE and dice	DSC = 0.908 HD = 5.573	DSC = 0.842 HD = 7.252			DSC = 0.795 HD = 19.437	2022	Liang et al. (2022b)		
	BraTS 2021			DSC = 0.926 HD = 3.738	DSC = 0.867 HD = 11.084			DSC = 0.826 HD = 17.531				
CNN-transformer combined model (BiTr-UNet)	BraTS 2021	Adam	-	DSC = 0.926 HD = 9.165	DSC = 0.935 HD = 8.200			DSC = 0.951 HD = 3.742	2022	Jia et al. (2021)		
Ensemble modified TransBTS and nnUNet	BraTS 2021	-	CE and dice	DSC = 0.928 HD = 4.930	DSC = 0.876 HD = 17.203			DSC = 0.879 HD = 10.426	2022	Dobko et al. (2021)		
Hybrid CNN-transformer model with regularization (SegTransVAE)	BraTS 2021	-	VAE and dice	DSC = 0.905 HD = 3.570	DSC = 0.926 HD = 5.840			DSC = 0.855 HD = 2.890	2022	Pham et al. (2022)		
Convolution-and-transformer network (COTRNet)	BraTS 2021	Adam	WCE and dice	DSC = 0.951 HD = 9.772	DSC = 0.961 HD = 15.560			DSC = 0.935 HD = 3.255	2022	Yang et al., 2021a		
Swin UNEt TRansformer (Swin UNETR)	BraTS 2021	-	soft dice	DSC = 0.926 HD = 5.831	DSC = 0.885 HD = 3.770			DSC = 0.858 HD = 6.016	2022	Futrega et al. (2021)		
Trans-NUNet	Kaggle	-	CE and dice	Avg. DSC = 0.864					2022	Wang et al., 2022a		
Efficient R-transformer network (ERTN)	BraTS 2017	AdamW	focal and dice	DSC = 0.832 HD = 5.300	DSC = 0.779 HD = 4.600			DSC = 0.726 HD = 5.500	2023	Hu et al. (2023)		
Deep semantics and edge information for brain tumor segmentation	BraTS 2018	Adam	BCE and dice	DSC = 0.909 HD = 3.923	DSC = 0.879 HD = 5.217			DSC = 0.819 HD = 3.440	2023	Zhu et al. (2023a)		
	BraTS 2019			DSC = 0.916 HD = 3.866	DSC = 0.892 HD = 5.118			DSC = 0.838 HD = 3.080				
	BraTS 2020			DSC = 0.910 HD = 4.719	DSC = 0.882 HD = 5.985			DSC = 0.846 HD = 3.051				
Deep mutual learning with fusion network for brain tumor segmentation	BraTS 2019	Adam	focal and active contour	DSC = 0.901 HD = 4.800	DSC = 0.840 HD = 6.112			DSC = 0.801 HD = 3.282	2023	Gao et al. (2023a)		
Flexible Fusion Network (F2 Net)	BraTS 2019	SGD	CE and dice	DSC = 0.950 HD = 2.21	DSC = 0.943 HD = 1.63			DSC = 0.902 HD = 1.33	2023	Yang et al. (2023)		
	BraTS 2020			DSC = 0.953 HD = 2.20	DSC = 0.945 HD = 1.59			DSC = 0.905 HD = 1.32				
Multi-scale ghost CNN with auxiliary MetaFormer decoding path (GMetaNet)	BraTS 2018	Adam	generalized dice	DSC = 0.901 HD = 5.16	DSC = 0.840 HD = 5.26			DSC = 0.820 HD = 2.62	2023	Lu et al. (2023)		
	BraTS 2019			DSC = 0.902 HD = 4.530	DSC = 0.825 HD = 6.400			DSC = 0.785 HD = 3.590				

## 3 Statistical analysis

In this section, we will delve into DL-based brain tumor segmentation models with an emphasis on statistical insights. To commence, we look at the data, particularly focusing on the number of papers published in the preceding 3 years, spanning from 2021 to 2023. This analysis provides valuable insights into current trends and achievements, offering a glimpse into the pace of evolution within the field. Subsequently, we explore the datasets commonly utilized by researchers in modern brain tumor segmentation studies. Understanding these datasets is essential since they give actual data for testing DL models. It is similar to inspecting the tools in a toolbox: the more we understand them, the more efficiently we can utilize them. Finally, we outline the assessment criteria commonly employed by researchers to evaluate the performance of DL models in the task of multimodal brain tumor segmentation. These metrics serve as benchmarks, enabling us to gauge the efficacy of these models accurately.

### 3.1 Publication statistics

**FIGURE 4:**
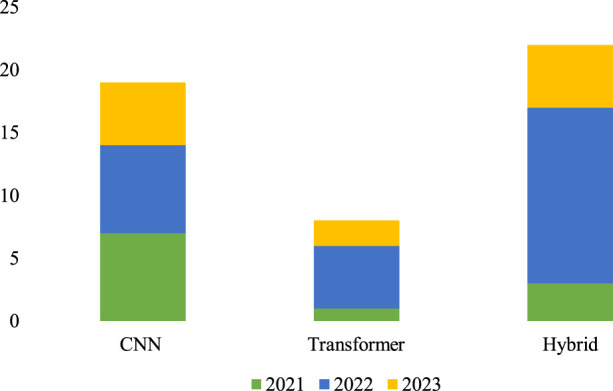
DL-based multi-modal MRI brain tumor segmentation model publication statistics from 2021 to 2023.

The field of brain tumor segmentation using DL models has seen tremendous advancements in recent years, with notable contributions from several architectures. In 2021, Dosovitskiy et al. (Dosovitskiy et al., 2020) introduced the vision transformer, which successfully applied the transformer architecture from natural language processing (NLP) to computer vision, marking a significant advancement. This pioneering research marked the beginning of the effective use of transformers in areas outside natural language processing (NLP), expanding into other computer vision tasks such as image classification, segmentation, and detection. Since the introduction of the vision transformer, the field of DL models has seen a significant increase in innovation, with the emergence of models that use transformers, CNNs, and hybrid architectures. This survey presents a thorough overview of brain tumor segmentation methodologies based on CNN, transformer, and hybrid models between 2021 and 2023. Figure 4 graphically represents the patterns in publications over this time, demonstrating the continuous shifts and diverse contributions from all these models.

### 3.2 Dataset statistics

The presence of multi-modal MRI datasets is indispensable for the effective evaluation of DL-based brain tumor segmentation models. Beginning in 2012, the Medical Imaging Computing and Computer-Aided Intervention Association (MICCAI) initiated the annual BraTS challenge. This longstanding challenge serves a pivotal role in fostering research and establishing a benchmark for evaluating brain tumor segmentation methods in the field. The BraTS challenge provides a standardized multi-modal MRI dataset consisting of four distinct scans - T1, T1, T2, and FLAIR. These modalities collectively offer a comprehensive view of brain anatomy and pathology, enabling researchers to develop and assess DL-based brain tumor segmentation methods. The influence of the BraTS challenge on research methodologies is profound, with a majority of studies opting to utilize BraTS datasets for training and testing their segmentation approaches.

**FIGURE 5:**
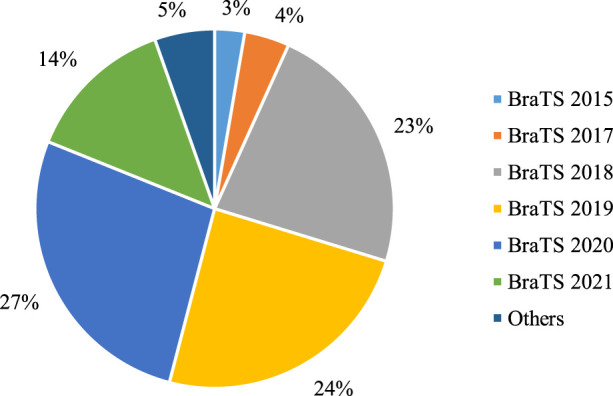
Statistical analysis of multi-modal brain tumor segmentation datasets used in the DL = based models from 2021 to 2023.

Figure 5 provides a quantitative analysis of the utilization of multi-modal MRI datasets in DL-based models over the past 3 years. Notably, over 97% of studies have leveraged BraTS datasets, with BraTS 2018, 2019, and 2020 emerging as the most commonly employed versions. While a few studies incorporate private datasets for segmentation performance comparisons, the prevailing trend emphasizes the use of publicly accessible BraTS datasets. The widespread availability and standardized nature of BraTS datasets make them the preferred choice, despite challenges posed by private datasets, such as the labor-intensive pixel-level annotations. As we anticipate future studies, the overarching trajectory is expected to continue toward the refinement and advancement of brain tumor segmentation methods utilizing the established and publicly accessible BraTS datasets. Table 7 provides the top BraTS databases. mostly used in the evaluation of brain tumor segmentation.

**TABLE 7:** Brain tumor segmentation (BraTS) datasets.

Dataset	Number of images	Available modalities
BraTS 2012	50	T1, T2, T1ce, FLAIR
BraTS 2013	60	T1, T2, T1ce, FLAIR
BraTS 2014	238	T1, T2, T1ce, FLAIR
BraTS 2015	253	T1, T2, T1ce, FLAIR
BraTS 2016	391	T1, T2, T1ce, FLAIR
BraTS 2017	477	T1, T2, T1ce, FLAIR
BraTS 2018	542	T1, T2, T1ce, FLAIR
BraTS 2019	651	T1, T2, T1ce, FLAIR
BraTS 2020	660	T1, T2, T1ce, FLAIR
BraTS 2021	2000	T1, T2, T1ce, FLAIR

### 3.3 Evaluation metrics

Evaluation metrics are quantitative measures used to assess the performance of a segmentation model, as they provide objective insights into how well a particular model performs compared to the ground truth. The segmentation model used a binary classification method in which each pixel belongs to either the tumorous or non-tumorous regions, usually represented as 1 and 0, respectively. From an input image, we obtained the segmentation results produced by the segmentation model and compared them with the ground truth created by experts. Numerous quantitative segmentation assessment metrics can be produced using the true positive (TP), false negative (FN), false positive (FP), and true negative (TN) metrics. A TP is an outcome in which the model correctly predicts a positive class, whereas an FP is an outcome in which the model incorrectly predicts a positive class. Similarly, TN is the outcome in which the model correctly predicts the negative class, whereas FN is the outcome in which the model incorrectly predicts the negative class. The most widely used evaluation metrics for segmentation tasks are the dice similarity coefficient (DSC), intersection over union (IoU), accuracy, precision, recall, and Hausdorff distance (HD).

Firstly, DSC represents the ratio of the overlapping region of predicted and ground truth over the total region. Mathematically, DSC is expressed as shown in Eq. (1):

$$DSC=\frac{2\cdot Y_{\text{pre}}\cap Y_{\text{GT}}}{Y_{\text{pre}}+Y_{\text{GT}}}$$ (1)

where $Y_{\text{pre}}$ represents the segmentation or predicted pixel and $Y_{\text{GT}}$ represents the groundtruth pixels.

Regarding the segmentation task, DSC is equal to the F1 score, and expressed in Eq. (2):

$$DSC=\frac{2\cdot TP}{2\cdot TP+FP+FN}$$ (2)

Then, IoU is a metric for quantifying the overlap between the segmentation prediction and the expert annotation (ground truth). This metric is defined as the proportion of the overlap between the segmentation outcome and the actual ground truth about their unions. The mathematical expression of IoU is formulated Eq. (3):

$$IoU=\frac{Y_{\text{pre}}\cap Y_{\text{GT}}}{Y_{\text{pre}}\cup Y_{\text{GT}}}$$ (3)

It is important to highlight that the Jaccard similarity coefficient and the IoU are equivalent. As a result, we may use TP, FP, and FN to rewrite the IoU expression as shown in Eq. (4):

$$IoU=\frac{TP}{TP+FP+FN}$$ (4)

Precision assesses the accuracy of positive predictions and is computed as the proportion of correctly identified positive outcomes relative to the combined total of true positives and false positives. It provides insight into the accuracy of positive predictions made by the model by indicating how many were correct. The mathematical expression for precision is shown in Eq. (5):

$$Precision=\frac{TP}{TP+FP}$$ (5)

Recall assesses the model’s capability to accurately identify all relevant positive instances by determining how many actual positive instances the model correctly identifies. and is computed as the proportion of correctly identified positive instances relative to the combined total of accurately identified positives and incorrectly identified negatives. The mathematical expression for recall is shown in Eq. (6):

$$Recall=\frac{TP}{TP+FN}$$ (6)

Accuracy is a comprehensive metric measuring the overall correctness of the model’s predictions, encompassing both positive and negative predictions. It is calculated as the sum of true positive and true negative results divided by the total number of predictions. Accuracy assesses how many predictions, both positive and negative, the model got correct out of all predictions made. The mathematical expression for accuracy is shown in Eq. (7):

$$Accuracy=\frac{TP+TN}{TP+FP+FN+TN}$$ (7)

HD serves as a distance-based evaluation metric. Within the HD, the predictions and the expert annotations are regarded as two distinct subsets in the measurement space. The mathematical expression is articulated in Eq. (8):

$$HD=\max\left(\underset{Y_{\text{pre}}}{\sup}\underset{Y_{\text{GT}}}{\inf}d\left(Y_{\text{pre}},Y_{\text{GT}}\right),\underset{Y_{\text{GT}}}{\sup}\underset{Y_{\text{pre}}}{\inf}d\left(Y_{\text{pre}},Y_{\text{GT}}\right)\right)$$ (8)

Here, “Sup” denotes the supremum, which is the least upper bound of a set, while “inf” signifies the infimum, which is the greatest lower bound of a set. So, $sup_{Y_{\text{pre}}}$ denotes the supremum over all possible subsets of $Y_{\text{pre}}$ and $inf_{Y_{\text{GT}}}$ denotes the infimum over all possible subsets of $Y_{\text{GT}}$ .

In summary, this section has extensively examined deep learning (DL)-based models for brain tumor segmentation, focusing on statistical insights. We began by scrutinizing the publication landscape from 2021 to 2023, revealing dynamic trends and the rapid evolution of DL-based approaches. Moving to dataset statistics, the indispensability of multi-modal MRI datasets, particularly through the MICCAI BraTS challenge, was emphasized. Figure 5 visually portrays the predominant use of BraTS datasets, underlining their widespread adoption. Additionally, we presented a comprehensive overview of common evaluation metrics, including DSC, IoU, accuracy, precision, recall, and HD, providing quantitative benchmarks for assessing model performance. Anticipating future studies, the trajectory is poised to continue refining brain tumor segmentation methods, leveraging established datasets and standardized metrics for ongoing advancements in this critical medical imaging domain.

## 4 Open research problem and possible future directions

DL-based segmentation of brain tumors using MRI images is a prominent area of research in medical imaging and has achieved good results. The diagnosis, therapy planning, and ongoing observation of people with tumors depend on precise segmentation. The development of the DL model for brain tumor segmentation is complex. In this section, we examine major research challenges that must be resolved.

### 4.1 Incomplete modalities

Incomplete modalities pose a significant challenge in medical image analysis. While numerous studies demonstrate impressive results when equipped with complete modalities, their efficacy diminishes when utilizing incomplete modalities as input sources (Azad et al., 2022). In practice, acquiring all modalities is often impractical, leading medical institutions to possess only partial modalities. Leveraging established methods for the segmentation of brain tumors across multiple modalities. Becomes challenging in such scenarios, hampering accurate diagnoses. In clinical practices, medical institutions frequently encounter incomplete MRI modalities due to limitations in collection devices.

Previous works in brain tumor segmentation typically assume complete input MRI data, resulting in a notable decline in performance when confronted with incomplete modality inputs. For instance, RFNet (Ding et al., 2021) shows the effect of missing modality using the BraTS 2020 dataset. They achieved a maximum DSC of 87.32% on WT using only the FLAIR modality, but with the combination of three modalities, i.e., FLAIR, T1, and T1ce, RFNet achieved a DSC of 90.69%. On the other hand, RFNet achieved 91.11% DSC using all four modalities. Similarly, in (Zhang et al., 2022b) mmformer achieved a DSC of 86.10%, 88.14%, and 89.64% using only FLAIR, three modalities (FlAIR, T1ce, and T2), and all four modalities using BraTS 2018.

Moreover, various recent models (Zhou et al., 2020; Wang et al., 2021c; Yang et al., 2022; Diao et al., 2023; Ting and Liu, 2023) were developed to handle the missing modalities effectively, but there remains some degradation in the performance compared to all modalities. To address this limitation, it is imperative to devise robust segmentation methods capable of handling incomplete modalities. Recently, some works have been proposed to tackle this issue (Zhao et al., 2022; Shi et al., 2023), however, most are tailored to specific cases of incomplete modalities and lack adaptability to diverse scenarios. Future research efforts should prioritize the development of a unified framework capable of robustly handling all cases, both complete and incomplete modalities alike.

### 4.2 Limited label data

A primary challenge in training the transformer model for segmentation is insufficiently labeled data. Medical images, particularly MRI datasets, possess an inadequate number of sample images compared to non-medical datasets. For example, the BraTS 2012 data from MICCAI challenges contain fewer images, as shown in Table 7. In contrast, non-medical datasets, such as ImageNet (Deng et al., 2009), contain over 1.2 million images, and the MNIST dataset (Deng et al., 2012) comprises 70,000 images. These limitations make transformer-based segmentation models less robust and generalizable for medical tasks because they require extensive and diverse datasets to understand the complicated and high-level properties of the tumor and its surrounding tissues.

The problem of limited data, specifically for brain tumors, can be tackled by using different augmentation techniques. These techniques generate training data and improve the model’s performance. Generally, there are two types of data augmentation, i.e., conventional and GAN-based. In conventional augmentation approaches, different transformations, such as geometric and photometric transformations, are used to increase the data quantity. However, these techniques are not effective in dealing with diverse data. On the other hand, GAN-based augmentation has gained popularity owing to its ability to produce synthetic and diverse data that closely resemble input data. A GAN is composed of a generator and discriminator neural networks. The generator network learns to create synthetic samples, whereas the discriminator network determines the differences between the actual and created samples.

Furthermore, researchers have employed various GAN-based data-augmentation techniques such as conditional GAN (cGAN) (Isola et al., 2017), cycle GAN (Sandfort et al., 2019), and parasitic GAN (Sun et al., 2019) for this purpose. Moreover, test-time augmentation methods can also be explored, as (Amiri et al., 2022) suggest that test-time augmentation (TTA) is one of the influential factors in improving model performance. The list of open-source packages and frameworks for DL-based medical image data augmentation methods are as follows: Augmentor (Bloice et al., 2019), Albumentations (Buslaev et al., 2020), Batchgenerators (Isensee et al., 2020), CutBlue (Yoo et al., 2020), CLoDSA (Casado-García et al., 2019), Gryds (Eppenhof and Pluim, 2018), ImgAug (Gu et al., 2019), Keras ImagedataGenerator (Chollet et al., 2015), MONAI (Cardoso et al., 2022), Pymia (Jungo et al., 2021), PyTorch Transformer (Paszke et al., 2019), and TorchIO (Pérez-García et al., 2021).

### 4.3 Enhancing model efficiency and deployment

In real-world scenarios, adeptly trained deep models find applications on terminal devices characterized by constrained resource availability. These settings’ requirements necessitate deploying efficient and lightweight deep models. During the training phase, emphasis is placed on ensuring the efficiency and compactness of deep models. Model compression strategies, including weight pruning, quantization, distillation of large models, and the incorporation of low-rank approximations, are employed to diminish the model’s size. These techniques effectively minimize the memory and computational demands of the deep model. Additionally, optimizing network architectures and implementing tailored training regimens contribute to alleviating the demand for an excessive number of parameters while maintaining optimal performance. Regrettably, there exists a dearth of research addressing the crucial aspects of model efficiency and deployment in brain tumor segmentation. This represents a pivotal gap in understanding that needs attention to ensure the successful utilization of algorithms in upcoming clinical practices.

### 4.4 Class imbalance

Addressing class imbalance is an essential challenge in the task of multi-modal brain tumor segmentation due to the tendency of these tumors to occupy a very limited area of the brain, which in turn complicates the processing of MRI data. The disparity might lead to a skewed division that favors the more significant class (healthy tissue), affecting the precise segmentation of brain tumors with smaller areas (Akil et al., 2020; Deepak and Ameer, 2023). Conventional methods include using class reweighting strategies throughout the training process to tackle this problem. These strategies provide more importance to the minority class (small tumor areas) and lesser significance to the majority class, allowing the model to prioritize the smaller class during training.

Recently, some work has been done to overcome the issue of class imbalance for multimodal MRI brain tumor segmentation. Most of the work in the literature is based on the use of different loss functions, for instance, in (Li et al., 2023b) combined loss function is used to optimize the network. Here dice and cross entropy losses are used to overcome class imbalance and stable training process, respectively. In (Zhu et al., 2023b), the edge detection module is for the class imbalance problem and they introduce $L_{\text{edge}}$ which is the combination of edge loss and dice. In (Yeung et al., 2022) introduce the unified focal loss for the class imbalance problem and also present the detailed discussion and effect of other loss functions. Various other loss functions are used in the literature to tackle the class imbalance problems (Jiao et al., 2023; Rehman et al., 2023; Li et al., 2024). Another way to deal with the class imbalance problem is to remove focus-free images in the dataset during training that are present in an excessive amount (Gao et al., 2023; Wu et al., 2024). Future research should focus on expanding current class balancing approaches and devise adaptive balancing approaches to improve the efficacy of tiny tumor areas.

### 4.5 Interpretability

Interpretability poses a difficulty in DL since these methods are often regarded as completely opaque models with limited insight into the reasoning behind predictions. The absence of interpretability is particularly critical in practical scenarios, notably in the field of clinical treatment, where understanding the functioning of deep models and the reasoning behind their choices is essential. One possible method to tackle this problem is using visual representations of feature maps, emphasizing prominent areas that influence the model’s results. Researchers have developed many techniques to display intermediate layers in deep learning models, such as activation maximization, class activation maps (Muhammad et al., 2020), and conditional t-distributed stochastic neighbor embedding (ct-SNE) (Kang et al., 2021). Recent endeavors have also used feature attribution techniques to identify the most relevant characteristics for a certain prediction generated by a DL model. The techniques involved in this process include gradient-based attribution (Ancona et al., 2017), perturbation-based attribution (Ivanovs et al., 2021) etc.

To further enhance the interpretability of multimodal DL models, eXplainable AI (XAI) offers a suite of techniques aimed at providing transparency and insights into model decisions. Some notable XAI methodologies include SHapley Additive exPlanations (SHAP) and Local Interpretable Model-agnostic Explanations (LIME). SHAP assesses the outcome for any DL model by determining the relative contributions of every feature to the resulting estimation and prediction, making it particularly useful for multimodal models (Lundberg et al., 2017). LIME explains individual predictions by approximating the model locally with an interpretable model. It achieves interpretability by training these models on subsets of the dataset, enabling users to understand how different features influence the decision (Ribeiro et al., 2016). Various other techniques have been developed for XAI, recently one method was developed for contrasting the decision-making patterns of the black box and white box models (Žlahtič et al., 2024).

Future research in multimodal brain tumor segmentation, utilizing Vision Transformers and other advanced architectures, provides the potential to customize interpretability approaches for individual purposes. By integrating these XAI techniques, we can improve the understanding of model functioning, increasing its transparency and offering useful insights for therapeutic applications. Emphasizing interpretability in multimodal DL models not only aids in clinical decision-making but also builds trust among medical professionals and patients, facilitating the adoption of AI technologies in healthcare.

## 5 Conclusion

This study highlights the significance of DL in brain tumor segmentation using multi-modal MRI, offering critical insights into treatment planning and personalized care. Beginning with exploring MRI modalities and the advantages of DL-based segmentation models. DL models have significantly improved brain tumor segmentation using multi-modal MRI and offered numerous advantages for tumor segmentation tasks, such as saving time, eliminating human bias, and minimizing errors. We thoroughly investigated DL-based models for brain tumor segmentation using multi-modal MRI and evaluated the recent existing model. Our study categorizes current research into three main groups based on the model’s architecture: CNN, transformer, and hybrid models. We have thoroughly investigated these models, considering their architectural design, dataset utilized, and experimental parameters. In addition, we perform a comprehensive statistical analysis of recent publications, brain tumor datasets, and evaluation metrics. Finally, open research challenges are identified and suggested promising future directions for multi-modal MRI brain tumor segmentation.

## References

[B1] Akbar A. S. Fatichah C. Suciati N. (2021). “Unet3d with multiple atrous convolutions attention block for brain tumor segmentation,” in International MICCAI brainlesion w orkshop (Springer), 182–193.

[B2] Akil M. Saouli R. Kachouri R. (2020). Fully automatic brain tumor segmentation with deep learning-based selective attention using overlapping patches and multi-class weighted cross-entropy. Med. image Anal. 63, 101692. 10.1016/j.media.2020.101692 32417714

[B3] Ali S. Li J. Pei Y. Khurram R. Rehman K. u. Mahmood T. (2022). A comprehensive survey on brain tumor diagnosis using deep learning and emerging hybrid techniques with multi-modal mr image. Archives Comput. methods Eng. 29, 4871–4896. 10.1007/s11831-022-09758-z

[B4] Amiri S. Ibragimov B. (2022). Improved automated lesion segmentation in whole-body fdg/pet-ct via test-time augmentation. arXiv Prepr. arXiv:2210.07761. 10.48550/arXiv.2210.07761

[B5] Ancona M. Ceolini E. Öztireli C. Gross M. (2017). Towards a better understanding of gradient-based attribution methods for deep neural networks. arXiv preprint arXiv:1711.06104.

[B6] Azad R. Khosravi N. Dehghanmanshadi M. Cohen-Adad J. Merhof D. (2022). Medical image segmentation on mri images with missing modalities: a review. arXiv preprint arXiv:2203.06217.

[B7] Biratu E. S. Schwenker F. Debelee T. G. Kebede S. R. Negera W. G. Molla H. T. (2021). Enhanced region growing for brain tumor mr image segmentation. J. Imaging 7, 22. 10.3390/jimaging7020022 34460621 PMC8321280

[B8] Bloice M. D. Roth P. M. Holzinger A. (2019). Biomedical image augmentation using augmentor. Bioinformatics 35, 4522–4524. 10.1093/bioinformatics/btz259 30989173

[B9] Buslaev A. Iglovikov V. I. Khvedchenya E. Parinov A. Druzhinin M. Kalinin A. A. (2020). Albumentations: fast and flexible image augmentations. Information 11, 125. 10.3390/info11020125

[B10] Cardoso M. J. Li W. Brown R. Ma N. Kerfoot E. Wang Y. (2022). Monai: an open-source framework for deep learning in healthcare. arXiv preprint arXiv:2211.02701.

[B11] Casado-García Áa Domínguez C. García-Domínguez M. Heras J. Inés A. Mata E. (2019). Clodsa: a tool for augmentation in classification, localization, detection, semantic segmentation and instance segmentation tasks. BMC Bioinforma. 20, 323. 10.1186/s12859-019-2931-1 PMC6567576 31195959

[B12] Çetiner H. Metlek S. (2023). Denseunet+: a novel hybrid segmentation approach based on multi-modality images for brain tumor segmentation. J. King Saud University-Computer Inf. Sci. 35, 101663. 10.1016/j.jksuci.2023.101663

[B13] Chang Y. Zheng Z. Sun Y. Zhao M. Lu Y. Zhang Y. (2023). Dpafnet: a residual dual-path attention-fusion convolutional neural network for multimodal brain tumor segmentation. Biomed. Signal Process. Control 79, 104037. 10.1016/j.bspc.2022.104037

[B14] Charles N. A. Holland E. C. Gilbertson R. Glass R. Kettenmann H. (2011). The brain tumor microenvironment. Glia 59, 1169–1180. 10.1002/glia.21136 21446047

[B15] Chen Y. Wang J. (2022). “Tseunet: a 3d neural network with fused transformer and se-attention for brain tumor segmentation,” in 2022 IEEE 35th international symposium on computer-based medical systems (CBMS) (IEEE), 131–136.

[B16] Chollet F. (2015). Keras: deep learning library for theano and tensorflow, T1. Available at: https://keras.io/k7.

[B17] Deepak S. Ameer P. (2023). Brain tumor categorization from imbalanced mri dataset using weighted loss and deep feature fusion. Neurocomputing 520, 94–102. 10.1016/j.neucom.2022.11.039

[B18] Deng J. Dong W. Socher R. Li L. J. Li K. Fei-Fei L. (2009). “Imagenet: a large-scale hierarchical image database,” in 2009 IEEE conference on computer vision and pattern recognition (Ieee), 248–255.

[B19] Deng L. (2012). The mnist database of handwritten digit images for machine learning research [best of the web]. IEEE signal Process. Mag. 29, 141–142. 10.1109/msp.2012.2211477

[B20] Desjardins L. Barrera M. Schulte F. Chung J. Cataudella D. Janzen L. (2019). Predicting social withdrawal, anxiety and depression symptoms in pediatric brain tumor survivors. J. Psychosoc. Oncol. 37, 22–36. 10.1080/07347332.2018.1535531 30614410

[B21] Dhole N. V. Dixit V. V. (2022). Review of brain tumor detection from mri images with hybrid approaches. Multimedia tools Appl. 81, 10189–10220. 10.1007/s11042-022-12162-1

[B22] Diao Y. Li F. Li Z. (2023). Joint learning-based feature reconstruction and enhanced network for incomplete multi-modal brain tumor segmentation. Comput. Biol. Med. 163, 107234. 10.1016/j.compbiomed.2023.107234 37450967

[B23] Ding Y. Yu X. Yang Y. (2021). “Rfnet: region-aware fusion network for incomplete multi-modal brain tumor segmentation,” in Proceedings of the IEEE/CVF international conference on computer vision, 3975–3984.

[B25] Dobko M. Kolinko D. I. Viniavskyi O. Yelisieiev Y. (2021). “Combining cnns with transformer for multimodal 3d mri brain tumor segmentation,” in International MICCAI brainlesion workshop (Springer), 232–241.

[B26] Dosovitskiy A. Beyer L. Kolesnikov A. Weissenborn D. Zhai X. Unterthiner T. (2020). An image is worth 16x16 words: transformers for image recognition at scale. *arXiv preprint arXiv:2010.11929* .

[B27] Eppenhof K. A. Pluim J. P. W. (2018). Pulmonary ct registration through supervised learning with convolutional neural networks. IEEE Trans. Med. imaging 38, 1097–1105. 10.1109/tmi.2018.2878316 30371358

[B28] Fang F. Yao Y. Zhou T. Xie G. Lu J. (2021). Self-supervised multi-modal hybrid fusion network for brain tumor segmentation. IEEE J. Biomed. Health Inf. 26, 5310–5320. 10.1109/jbhi.2021.3109301 34478389

[B29] Futrega M. Milesi A. Marcinkiewicz M. Ribalta P. (2021). “Optimized u-net for brain tumor segmentation,” in International MICCAI brainlesion workshop (Springer), 15–29.

[B30] Gao H. Miao Q. Ma D. Liu R. (2023a). Deep mutual learning for brain tumor segmentation with the fusion network. Neurocomputing 521, 213–220. 10.1016/j.neucom.2022.11.038

[B31] Gao L. Li J. Zhang R. Bekele H. H. Wang J. Cheng Y. (2023b). Mmgan: a multimodal mr brain tumor image segmentation method. Front. Hum. Neurosci. 17, 1275795. 10.3389/fnhum.2023.1275795 38116237 PMC10728273

[B32] Gu S. Bao J. Yang H. Chen D. Wen F. Yuan L. (2019). “Mask-guided portrait editing with conditional gans,” in Proceedings of the IEEE/CVF conference on computer vision and pattern recognition, 3436–3445.

[B33] Guo X. Yang C. Lam P. L. Woo P. Y. Yuan Y. (2020). “Domain knowledge-based brain tumor segmentation and overall survival prediction,” in Brainlesion: glioma, multiple sclerosis, stroke and traumatic brain injuries: 5th international workshop, BrainLes 2019, held in conjunction with MICCAI 2019, shenzhen, China, october 17, 2019 (Springer), 285–295.

[B34] Hamamci A. Unal G. (2012). Multimodal brain tumor segmentation using the tumor-cut method on the brats dataset. Proc. MICCAI-BraTS, 19–23.

[B35] Hamamci A. Kucuk N. Karaman K. Engin K. Unal G. (2011). Tumor-cut: segmentation of brain tumors on contrast-enhanced mr images for radiosurgery applications. IEEE Trans. Med. imaging 31, 790–804. 10.1109/tmi.2011.2181857 22207638

[B36] He W. Zhou X. Mao Y. Wu Y. Tang X. Yan S. (2022). Circcrim1 promotes nasopharyngeal carcinoma progression via the mir-34c-5p/fosl1 axis. Eur. J. Med. Res. 27, 59. 10.1186/s40001-022-00667-2 35484574 PMC9052594

[B37] Hou Q. Peng Y. Wang Z. Wang J. Jiang J. (2023). Mfd-net: modality fusion diffractive network for segmentation of multimodal brain tumor image. IEEE J. Biomed. Health Inf. 27, 5958–5969. 10.1109/jbhi.2023.3318640 37747864

[B38] Hu Z. Li L. Sui A. Wu G. Wang Y. Yu J. (2023). An efficient r-transformer network with dual encoders for brain glioma segmentation in mr images. Biomed. Signal Process. Control 79, 104034. 10.1016/j.bspc.2022.104034

[B39] Huang L. Zhu E. Chen L. Wang Z. Chai S. Zhang B. (2022b). A transformer-based generative adversarial network for brain tumor segmentation. Front. Neurosci. 16, 1054948. 10.3389/fnins.2022.1054948 36532274 PMC9750177

[B40] Huang Z. Lin L. Cheng P. Peng L. Tang X. (2022a). Multi-modal brain tumor segmentation via missing modality synthesis and modality-level attention fusion. arXiv preprint arXiv:2203.04586.

[B41] Ilhan U. Ilhan A. (2017). Brain tumor segmentation based on a new threshold approach. Procedia Comput. Sci. 120, 580–587. 10.1016/j.procs.2017.11.282

[B42] Isensee F. Jäger P. Wasserthal J. Zimmerer D. Petersen J. Kohl S. (2020). batchgenerators—a python framework for data augmentation. Zenodo 3632567.

[B43] Isola P. Zhu J. Y. Zhou T. Efros A. A. (2017). “Image-to-image translation with conditional adversarial networks,” in Proceedings of the IEEE conference on computer vision and pattern recognition, 1125–1134.

[B44] Ivanovs M. Kadikis R. Ozols K. (2021). Perturbation-based methods for explaining deep neural networks: a survey. Pattern Recognit. Lett. 150, 228–234. 10.1016/j.patrec.2021.06.030

[B45] Jia Q. Shu H. (2021). “Bitr-unet: a cnn-transformer combined network for mri brain tumor segmentation,” in International MICCAI brainlesion workshop (Springer), 3–14. 10.1007/978-3-031-09002-8_1 PMC9396958 36005929

[B46] Jiao C. Yang T. Yan Y. Yang A. (2023). Rftnet: region–attention fusion network combined with dual-branch vision transformer for multimodal brain tumor image segmentation. Electronics 13, 77. 10.3390/electronics13010077

[B47] Jun E. Jeong S. Heo D. W. Suk H. I. (2021). Medical transformer: universal brain encoder for 3d mri analysis. arXiv preprint arXiv:2104.13633. 10.1109/TNNLS.2023.3308712 37738193

[B48] Jungo A. Scheidegger O. Reyes M. Balsiger F. (2021). pymia: a python package for data handling and evaluation in deep learning-based medical image analysis. Comput. methods programs Biomed. 198, 105796. 10.1016/j.cmpb.2020.105796 33137700

[B49] Jyothi P. Singh A. R. (2023). Deep learning models and traditional automated techniques for brain tumor segmentation in mri: a review. Artif. Intell. Rev. 56, 2923–2969. 10.1007/s10462-022-10245-x

[B50] Kang B. García García D. Lijffijt J. Santos-Rodríguez R. De Bie T. (2021). Conditional t-sne: more informative t-sne embeddings. Mach. Learn. 110, 2905–2940. 10.1007/s10994-020-05917-0 34840420 PMC8599264

[B51] Karimijafarbigloo S. Azad R. Kazerouni A. Ebadollahi S. Merhof D. (2023). “Mmcformer: missing modality compensation transformer for brain tumor segmentation,” in Medical imaging with deep learning.

[B52] Khilkhal R. Ismael M. (2022). “Brain tumor segmentation utilizing thresholding and k-means clustering,” in 2022 muthanna international conference on engineering science and Technology (MICEST) (IEEE), 43–48.

[B53] Kotia J. Kotwal A. Bharti R. (2020). “Risk susceptibility of brain tumor classification to adversarial attacks,” in Man-machine interactions 6: 6th international conference on man-machine interactions, ICMMI 2019, cracow, Poland, october 2-3, 2019. Springer, 181–187.

[B54] Li D. Chen G. Wu X. Yu Z. Tan M. (2024a). Face anti-spoofing with cross-stage relation enhancement and spoof material perception. Neural Netw. 175, 106275. 10.1016/j.neunet.2024.106275 38653078

[B55] Li J. Wang W. Chen C. Zhang T. Zha S. Wang J. (2022). Transbtsv2: towards better and more efficient volumetric segmentation of medical images. arXiv preprint arXiv:2201.12785.

[B56] Li P. Li Z. Wang Z. Li C. Wang M. (2024b). mresu-net: multi-scale residual u-net-based brain tumor segmentation from multimodal mri. Med. Biol. Eng. Comput. 62, 641–651. 10.1007/s11517-023-02965-1 37981627

[B57] Li S. Sui X. Luo X. Xu X. Liu Y. Goh R. (2021). Medical image segmentation using squeeze-and-expansion transformers. arXiv Prepr. arXiv:2105.09511. 10.48550/arXiv.2105.09511

[B58] Li X. Jiang Y. Li M. Zhang J. Yin S. Luo H. (2023a). Msfr-net: multi-modality and single-modality feature recalibration network for brain tumor segmentation. Med. Phys. 50, 2249–2262. 10.1002/mp.15933 35962724

[B59] Li X. Jiang Y. Li M. Zhang J. Yin S. Luo H. (2023b). Msfr-net: multi-modality and single-modality feature recalibration network for brain tumor segmentation. Med. Phys. 50, 2249–2262. 10.1002/mp.15933 35962724

[B60] Liang J. Yang C. Zeng L. (2022b). 3d pswinbts: an efficient transformer-based unet using 3d parallel shifted windows for brain tumor segmentation. Digit. Signal Process. 131, 103784. 10.1016/j.dsp.2022.103784

[B61] Liang J. Yang C. Zeng M. Wang X. (2022a). Transconver: transformer and convolution parallel network for developing automatic brain tumor segmentation in mri images. Quantitative Imaging Med. Surg. 12, 2397–2415. 10.21037/qims-21-919 PMC8923874 35371952

[B62] Liu J. Zheng J. Jiao G. (2022d). Transition net: 2d backbone to segment 3d brain tumor. Biomed. Signal Process. Control 75, 103622. 10.1016/j.bspc.2022.103622

[B63] Liu Y. Mu F. Shi Y. Cheng J. Li C. Chen X. (2022b). Brain tumor segmentation in multimodal mri via pixel-level and feature-level image fusion. Front. Neurosci. 16, 1000587. 10.3389/fnins.2022.1000587 36188482 PMC9515796

[B64] Liu Z. Lv Q. Yang Z. Li Y. Lee C. H. Shen L. (2022a). Medical image analysis based on transformer: a review. arXiv preprint arXiv:2208.06643. 10.1016/j.compbiomed.2023.107268 37494821

[B65] Liu Z. Wei J. Li R. Zhou J. (2022c). Tfusion: transformer based n-to-one multimodal fusion block. arXiv preprint arXiv:2208.12776.

[B66] Liu Z. Tong L. Chen L. Jiang Z. Zhou F. Zhang Q. (2023). Deep learning-based brain tumor segmentation: a survey. Complex and intelligent Syst. 9, 1001–1026. 10.1007/s40747-022-00815-5

[B67] Lu Y. Chang Y. Zheng Z. Sun Y. Zhao M. Yu B. (2023). Gmetanet: multi-scale ghost convolutional neural network with auxiliary metaformer decoding path for brain tumor segmentation. Biomed. Signal Process. Control 83, 104694. 10.1016/j.bspc.2023.104694

[B68] Lundberg S. M. Lee S. I. (2017). A unified approach to interpreting model predictions. Adv. neural Inf. Process. Syst. 30.

[B69] Menze B. H. Jakab A. Bauer S. Kalpathy-Cramer J. Farahani K. Kirby J. (2014). The multimodal brain tumor image segmentation benchmark (brats). IEEE Trans. Med. imaging 34, 1993–2024. 10.1109/tmi.2014.2377694 25494501 PMC4833122

[B70] Mohammed Y. M. El Garouani S. Jellouli I. (2023). A survey of methods for brain tumor segmentation-based mri images. J. Comput. Des. Eng. 10, 266–293. 10.1093/jcde/qwac141

[B71] Muhammad M. B. Yeasin M. (2020). “Eigen-cam: class activation map using principal components,” in 2020 international joint conference on neural networks (IJCNN) (IEEE), 1–7.

[B72] Nyo M. T. Mebarek-Oudina F. Hlaing S. S. Khan N. A. (2022). Otsu’s thresholding technique for mri image brain tumor segmentation. Multimedia tools Appl. 81, 43837–43849. 10.1007/s11042-022-13215-1

[B73] Oktay O. Schlemper J. Folgoc L. L. Lee M. Heinrich M. Misawa K. (2018). Attention u-net: learning where to look for the pancreas. *arXiv preprint arXiv:1804.03999* .

[B74] Paszke A. Gross S. Massa F. Lerer A. Bradbury J. Chanan G. (2019). Pytorch: an imperative style, high-performance deep learning library. Adv. neural Inf. Process. Syst. 32.

[B75] Peiris H. Hayat M. Chen Z. Egan G. Harandi M. (2022a). “A robust volumetric transformer for accurate 3d tumor segmentation,” in International conference on medical image computing and computer-assisted intervention (Springer), 162–172.

[B76] Peiris H. Hayat M. Chen Z. Egan G. Harandi M. (2022b). Hybrid window attention based transformer architecture for brain tumor segmentation. arXiv preprint arXiv:2209.07704.

[B77] Pereira S. Pinto A. Alves V. Silva C. A. (2016). Brain tumor segmentation using convolutional neural networks in mri images. IEEE Trans. Med. imaging 35, 1240–1251. 10.1109/tmi.2016.2538465 26960222

[B78] Pérez-García F. Sparks R. Ourselin S. (2021). Torchio: a python library for efficient loading, preprocessing, augmentation and patch-based sampling of medical images in deep learning. Comput. Methods Programs Biomed. 208, 106236. 10.1016/j.cmpb.2021.106236 34311413 PMC8542803

[B79] Pham Q. D. Nguyen-Truong H. Phuong N. N. Nguyen K. N. Nguyen C. D. Bui T. (2022). “Segtransvae: hybrid cnn-transformer with regularization for medical image segmentation,” in 2022 IEEE 19th international symposium on biomedical imaging (ISBI) (IEEE), 1–5.

[B80] Philip A. K. Samuel B. Bhatia S. Khalifa S. El-Seedi H. (2022). Artificial intelligence and precision medicine: a new frontier for the treatment of brain tumors. Life 13, 24. 10.3390/life13010024 36675973 PMC9866715

[B81] Pinaya W. H. Tudosiu P. D. Gray R. Rees G. Nachev P. Ourselin S. (2022). Unsupervised brain imaging 3d anomaly detection and segmentation with transformers. Med. Image Anal. 79, 102475. 10.1016/j.media.2022.102475 35598520 PMC10108352

[B82] Privitera L. Paraboschi I. Dixit D. Arthurs O. J. Giuliani S. (2022). Image-guided surgery and novel intraoperative devices for enhanced visualisation in general and paediatric surgery: a review. Innov. Surg. Sci. 6, 161–172. 10.1515/iss-2021-0028 35937852 PMC9294338

[B83] Ranjbarzadeh R. Caputo A. Tirkolaee E. B. Jafarzadeh Ghoushchi S. Bendechache M. (2023). Brain tumor segmentation of mri images: a comprehensive review on the application of artificial intelligence tools. Comput. Biol. Med. 152, 106405. 10.1016/j.compbiomed.2022.106405 36512875

[B84] Rao C. S. Karunakara K. (2021). A comprehensive review on brain tumor segmentation and classification of mri images. Multimedia Tools Appl. 80, 17611–17643. 10.1007/s11042-020-10443-1

[B85] Rehman M. U. Ryu J. Nizami I. F. Chong K. T. (2023). Raagr2-net: a brain tumor segmentation network using parallel processing of multiple spatial frames. Comput. Biol. Med. 152, 106426. 10.1016/j.compbiomed.2022.106426 36565485

[B86] Ribeiro M. T. Singh S. Guestrin C. (2016). “Why should i trust you? explaining the predictions of any classifier,” in Proceedings of the 22nd ACM SIGKDD international conference on knowledge discovery and data mining, 1135–1144.

[B87] Ronneberger O. Fischer P. Brox T. (2015). “U-net: convolutional networks for biomedical image segmentation,” in Medical image computing and computer-assisted intervention–MICCAI 2015: 18th international conference, Munich, Germany, october 5-9, 2015, proceedings, Part III 18 (Springer), 234–241.

[B88] Sagar A. (2021a). “Vitbis: vision transformer for biomedical image segmentation,” in MICCAI workshop on distributed and collaborative learning (Springer), 34–45.

[B89] Sagar A. (2021b). “Emsvit: efficient multi scale vision transformer for biomedical image segmentation,” in International MICCAI brainlesion workshop (Springer), 39–51.

[B90] Sahoo A. K. Parida P. Muralibabu K. Dash S. (2023). “An improved dnn with ffcm method for multimodal brain tumor segmentation,” in Intelligent systems with applications.

[B91] Salvador R. Canales-Rodríguez E. Guerrero-Pedraza A. Sarró S. Tordesillas-Gutiérrez D. Maristany T. (2019). Multimodal integration of brain images for mri-based diagnosis in schizophrenia. Front. Neurosci. 13, 1203. 10.3389/fnins.2019.01203 31787874 PMC6855131

[B92] Sandfort V. Yan K. Pickhardt P. J. Summers R. M. (2019). Data augmentation using generative adversarial networks (cyclegan) to improve generalizability in ct segmentation tasks. Sci. Rep. 9, 16884. 10.1038/s41598-019-52737-x 31729403 PMC6858365

[B93] Shi J. Yu L. Cheng Q. Yang X. Cheng K. T. Yan Z. (2023). “M $\{2\}$ ftrans: modality-masked fusion transformer for incomplete multi-modality brain tumor segmentation,” in IEEE journal of biomedical and health informatics. 10.1109/JBHI.2023.3326151 37862279

[B94] Siegel R. L. Miller K. D. Wagle N. S. Jemal A. (2023). Cancer statistics, 2023. Ca Cancer J. Clin. 73, 17–48. 10.3322/caac.21763 36633525

[B95] Singha A. Thakur R. S. Patel T. (2021). “Deep learning applications in medical image analysis,” in Techniques and applications biomedical data mining for information retrieval: methodologies, 293–350. 10.1002/9781119711278.ch11

[B96] Stiles J. Jernigan T. L. (2010). The basics of brain development. Neuropsychol. Rev. 20, 327–348. 10.1007/s11065-010-9148-4 21042938 PMC2989000

[B97] Sun Y. Zhou Fu Y. Xue X. (2019). “Parasitic gan for semi-supervised brain tumor segmentation,” in 2019 IEEE international conference on image processing (ICIP) (IEEE), 1535–1539.

[B98] Syazwany N. S. Nam J. H. Lee S. C. (2021). Mm-bifpn: multi-modality fusion network with bi-fpn for mri brain tumor segmentation. IEEE Access 9, 160708–160720. 10.1109/access.2021.3132050

[B99] Tahir A. Asif M. Ahmad M. B. Mahmood T. Khan M. A. Ali M. (2022). Brain tumor detection using decision-based fusion empowered with fuzzy logic. Math. Problems Eng. 2022, 1–13. 10.1155/2022/2710285

[B100] Tian W. Lv M. Huang P. (2022). Axial attention convolutional neural network for brain tumor segmentation with multi-modality mri scans. Brain Sci. 13, 12. 10.3390/brainsci13010012 36671994 PMC9856007

[B101] Ting H. Liu M. (2023). Multimodal transformer of incomplete mri data for brain tumor segmentation. IEEE J. Biomed. Health Inf. 28, 89–99. 10.1109/jbhi.2023.3286689 37327094

[B102] Tong J. Wang C. (2023). A dual tri-path cnn system for brain tumor segmentation. Biomed. Signal Process. Control 81, 104411. 10.1016/j.bspc.2022.104411

[B103] Wang E. Hu Y. Yang X. Tian X. (2022a). “Transunet with attention mechanism for brain tumor segmentation on mr images,” in 2022 IEEE international conference on artificial intelligence and computer applications (ICAICA) (IEEE), 573–577.

[B104] Wang P. Liu S. Peng J. (2022b). “Ast-net: lightweight hybrid transformer for multimodal brain tumor segmentation,” in 2022 26th international conference on pattern recognition (ICPR) (IEEE), 4623–4629.

[B105] Wang P. Yang Q. He Z. Yuan Y. (2023a). Vision transformers in multi-modal brain tumor mri segmentation: a review. Meta-Radiology 1, 100004. 10.1016/j.metrad.2023.100004

[B106] Wang P. Yang Q. He Z. Yuan Y. (2023b). Vision transformers in multi-modal brain tumor mri segmentation: a review. Meta-Radiology 1, 100004. 10.1016/j.metrad.2023.100004

[B108] Wang Y. Chen J. Bai X. (2023c). Gradient-assisted deep model for brain tumor segmentation by multi-modality mri volumes. Biomed. Signal Process. Control 85, 105066. 10.1016/j.bspc.2023.105066

[B107] Wenxuan W. Chen C. Meng D. Hong Y. Sen Z. Jiangyun L. (2021a). “Transbts: multimodal brain tumor segmentation using transformer,” in Medical image computing and computer assisted intervention–MICCAI 2021: 24th international conference, strasbourg, France, september 27–october 1, 2021, proceedings, Part I 24 (Springer), 109–119.

[B109] Wang Y. Zhang Y. Hou F. Liu Y. Tian J. Zhong C. (2021b). “Modality-pairing learning for brain tumor segmentation,” in Brainlesion: glioma, multiple sclerosis, stroke and traumatic brain injuries: 6th international workshop, BrainLes 2020, held in conjunction with MICCAI 2020, Lima, Peru (Springer), 230–240.

[B110] Wang Y. Zhang Y. Liu Y. Lin Z. Tian J. Zhong C. (2021c). “Acn: adversarial co-training network for brain tumor segmentation with missing modalities,” in Medical image computing and computer assisted intervention–MICCAI 2021: 24th international conference, strasbourg, France, september 27–october 1, 2021, proceedings, Part VII 24 (Springer), 410–420.

[B111] Wu X. Yang X. Li Z. Liu L. Xia Y. (2024). Multimodal brain tumor image segmentation based on densenet. Plos one 19, e0286125. 10.1371/journal.pone.0286125 38236898 PMC10796062

[B112] Xing Z. Yu L. Wan L. Han T. Zhu L. (2022). “Nestedformer: nested modality-aware transformer for brain tumor segmentation,” in International conference on medical image computing and computer-assisted intervention (Springer), 140–150.

[B113] Yang H. Shen Z. Li Z. Liu J. Xiao J. (2021a). “Combining global information with topological prior for brain tumor segmentation,” in International MICCAI brainlesion workshop (Springer), 204–215.

[B114] Yang H. Zhou T. Zhou Y. Zhang Y. Fu H. (2023). Flexible fusion network for multi-modal brain tumor segmentation. IEEE J. Biomed. Health Inf. 27, 3349–3359. 10.1109/jbhi.2023.3271808 37126623

[B115] Yang Q. Yuan Y. (2021b). “Learning dynamic convolutions for multi-modal 3d mri brain tumor segmentation,” in Brainlesion: glioma, multiple sclerosis, stroke and traumatic brain injuries: 6th international workshop, BrainLes 2020, held in conjunction with MICCAI 2020, Lima, Peru, october 4, 2020 (Springer), 441–451.

[B116] Yang Q. Guo X. Chen Z. Woo P. Y. M. Yuan Y. (2022). D 2-net: dual disentanglement network for brain tumor segmentation with missing modalities. IEEE Trans. Med. Imaging 41, 2953–2964. 10.1109/tmi.2022.3175478 35576425

[B117] Yeung M. Sala E. Schönlieb C. B. Rundo L. (2022). Unified focal loss: generalising dice and cross entropy-based losses to handle class imbalanced medical image segmentation. Comput. Med. Imaging Graph. 95, 102026. 10.1016/j.compmedimag.2021.102026 34953431 PMC8785124

[B118] Yoo J. Ahn N. Sohn K. A. (2020). “Rethinking data augmentation for image super-resolution: a comprehensive analysis and a new strategy,” in Proceedings of the IEEE/CVF conference on computer vision and pattern recognition, 8375–8384.

[B119] Zhang D. Huang G. Zhang Q. Han J. Han J. Yu Y. (2021). Cross-modality deep feature learning for brain tumor segmentation. Pattern Recognit. 110, 107562. 10.1016/j.patcog.2020.107562

[B120] Zhang J. Qin Q. Ye Q. Ruan T. (2023). St-unet: Swin transformer boosted u-net with cross-layer feature enhancement for medical image segmentation. Comput. Biol. Med. 153, 106516. 10.1016/j.compbiomed.2022.106516 36628914

[B121] Zhang Y. He N. Yang J. Li Y. Wei D. Huang Y. (2022a). “mmformer: multimodal medical transformer for incomplete multimodal learning of brain tumor segmentation,” in International conference on medical image computing and computer-assisted intervention (Springer), 107–117.

[B123] Zhao L. Ma J. Shao Y. Jia C. Zhao J. Yuan H. (2022a). Mm-unet: a multimodality brain tumor segmentation network in mri images. Front. Oncol. 12, 950706. 10.3389/fonc.2022.950706 36059677 PMC9434799

[B124] Zhao Z. Yang H. Sun J. (2022b). “Modality-adaptive feature interaction for brain tumor segmentation with missing modalities,” in International conference on medical image computing and computer-assisted intervention (Springer), 183–192.

[B125] Zhou T. Canu S. Vera P. Ruan S. (2021). Feature-enhanced generation and multi-modality fusion-based deep neural network for brain tumor segmentation with missing mr modalities. Neurocomputing 466, 102–112. 10.1016/j.neucom.2021.09.032

[B126] Zhou T. Canu S. Vera P. Ruan S. (2020). “Brain tumor segmentation with missing modalities via latent multi-source correlation representation,” in Medical image computing and computer assisted intervention–MICCAI 2020: 23rd international conference, Lima, Peru, october 4–8, 2020, proceedings, Part IV 23 (Springer), 533–541.

[B127] Zhou T. (2023). Modality-level cross-connection and attentional feature fusion-based deep neural network for multi-modal brain tumor segmentation. Biomed. Signal Process. Control 81, 104524. 10.1016/j.bspc.2022.104524

[B128] Zhou Z. Rahman Siddiquee M. M. Tajbakhsh N. Liang J. (2018). “Unet++: a nested u-net architecture for medical image segmentation,” in Deep learning in medical image analysis and multimodal learning for clinical decision support: 4th international workshop, DLMIA 2018, and 8th international workshop, ML-CDS 2018, held in conjunction with MICCAI 2018, granada, Spain, september 20, 2018, proceedings 4. Springer, 3–11. 10.1007/978-3-030-00889-5_1 PMC7329239 32613207

[B129] Zhu Z. He X. Qi G. Li Y. Cong B. Liu Y. (2023a). Brain tumor segmentation based on the fusion of deep semantics and edge information in multimodal mri. Inf. Fusion 91, 376–387. 10.1016/j.inffus.2022.10.022

[B130] Zhu Z. He X. Qi G. Li Y. Cong B. Liu Y. (2023b). Brain tumor segmentation based on the fusion of deep semantics and edge information in multimodal mri. Inf. Fusion 91, 376–387. 10.1016/j.inffus.2022.10.022

[B131] Žlahtič B. Završnik J. Blažun Vošner H. Kokol P. (2024). Transferring black-box decision making to a white-box model. Electronics 13, 1895. 10.3390/electronics13101895

